# Epigenomic, genomic, and transcriptomic landscape of schwannomatosis

**DOI:** 10.1007/s00401-020-02230-x

**Published:** 2020-10-06

**Authors:** Sheila Mansouri, Suganth Suppiah, Yasin Mamatjan, Irene Paganini, Jeffrey C. Liu, Shirin Karimi, Vikas Patil, Farshad Nassiri, Olivia Singh, Yogi Sundaravadanam, Prisni Rath, Roberta Sestini, Francesca Gensini, Sameer Agnihotri, Jaishri Blakeley, Kimberly Ostrow, David Largaespada, Scott R. Plotkin, Anat Stemmer-Rachamimov, Marcela Maria Ferrer, Trevor J. Pugh, Kenneth D. Aldape, Laura Papi, Gelareh Zadeh

**Affiliations:** 1 Princess Margaret Cancer Center and MacFeeters-Hamilton Center for Neuro-Oncology Research, University Health Network, Wilkins Family Chair in Brain Tumor Research, 14-701 PMCRT, 101 College St, Toronto, ON M5G 1L7 Canada; 2 The Department of Experimental and Clinical, Medical Genetics Unit, Biomedical Sciences “Mario Serio”, University of Florence, Florence, Italy; 3 Ontario Institute for Cancer Research, Toronto, ON Canada; 4 Department of Neurological Surgery, Children’s Hospital, University of Pittsburgh, Pittsburgh, PA USA; 5 Johns Hopkins University, Baltimore, MD USA; 6 Department of Paediatrics, University of MN, Minneapolis, USA; 7 Department of Pathology, Massachusetts General Hospital, Boston, MA USA; 8 División de Neurocirugía and División Genética, Hospital de Clínicas “José de San Martín”, Universidad de Buenos Aires, Buenos Aires, Argentina; 9 Laboratory of Pathology, Center for Cancer Research, National Cancer Institute, Bethesda, MD USA; 10 Division of Neurosurgery, Toronto Western Hospital, Toronto, Canada; 11 Krembil Brain Institute, Toronto, Canada

**Keywords:** Schwannomatosis, Peripheral nerve sheath tumors, LZTR1, Genomics, Pain, MAPK

## Abstract

Schwannomatosis (SWNTS) is a genetic cancer predisposition syndrome that manifests as multiple and often painful neuronal tumors called schwannomas (SWNs). While germline mutations in *SMARCB1* or *LZTR1,* plus somatic mutations in *NF2* and loss of heterozygosity in chromosome 22q have been identified in a subset of patients, little is known about the epigenomic and genomic alterations that drive SWNTS-related SWNs (SWNTS-SWNs) in a majority of the cases. We performed multiplatform genomic analysis and established the molecular signature of SWNTS-SWNs. We show that SWNTS-SWNs harbor distinct genomic features relative to the histologically identical non-syndromic sporadic SWNs (NS-SWNS). We demonstrate the existence of four distinct DNA methylation subgroups of SWNTS-SWNs that are associated with specific transcriptional programs and tumor location. We show several novel recurrent non-22q deletions and structural rearrangements. We detected the *SH3PXD2A-HTRA1* gene fusion in SWNTS-SWNs, with predominance in *LZTR1*-mutant tumors. In addition, we identified specific genetic, epigenetic, and actionable transcriptional programs associated with painful SWNTS-SWNs including PIGF, VEGF, MEK, and MTOR pathways, which may be harnessed for management of this syndrome.

**Electronic supplementary material:**

The online version of this article (10.1007/s00401-020-02230-x) contains supplementary material, which is available to authorized users.

## Introduction

Schwannomatosis (SWNTS; MIM #162091) is a genetic cancer-predisposing syndrome and a form of neurofibromatosis (NF) that affects approximately 1 in 126,315 individuals [11] and is characterized by the development of multiple non-intradermal schwannomas (SWNs), mainly in the peripheral nerves (90%) and spinal nerves (75%), and, less commonly, cranial nerves [30]. A hallmark of SWNTS is severe chronic localized or diffuse pain that negatively impacts patients’ quality of life and often leads to death. While the majority of SWNTS in the general population occur sporadically, 13–25% are associated with the autosomal dominant tumor suppressor SWNTS or NF3 syndrome with reduced penetrance [3, 28, 30].

While SWNs that occur in SWNTS are phenotypically and histopathologically indistinguishable from those that develop in NF2 disease and the non-syndromic SWNs (NS-SWNs), early evidence indicates that the molecular pathways and drivers of these tumors are very different. Unlike in NF2 disease, few genetic studies on SWNTS ruled out germline mutations in *NF2* gene in this disease [23] while somatic mutations in *NF2* gene and loss of heterozygosity (LOH) in chromosome 22q are the only recurrent somatic alterations that have been reported and confirmed in SWNTS-related SWNs (SWNTS-SWNs) [22]. Germline mutations in *SMARCB1* are found in 48% of familial and 10% of sporadic SWNTS [6, 18, 19, 32, 33, 40–43], while germline *LZTR1* mutations are found in 38% of familial and 30% of sporadic SWNTS. Thus, the molecular drivers of tumor formation in a large proportion of SWNTS-SWNs remain unknown.

Here, we have established the comprehensive molecular landscape, including DNA methylome, whole exome, whole genome, and transcriptome of the largest cohort of SWNTS-SWNs, and compare to NS-SWNs to identify the distinct molecular pathways and drivers responsible for these phenotypically similar tumors. This also serves as the largest dataset for mining specific molecular vulnerabilities that may be targeted for management of tumor burden and pain in SWNTS.

## Methods

### Cohort summary

Complete clinical and molecular profiling information for the SWNTS cohort can be found in Online Resource Table 1. Samples were subjected to pathology reassessment of hematoxylin and eosin-stained slides of tumor samples by 3 independent neuropathologists (ASR/KDA/SK) to confirm the diagnosis, determine histological subtype, and assess tumor purity. In addition, we performed DNA methylation profiling on 90 neurofibromas (56 plexiform, 34 cutaneous).

### DNA methylation profiling

DNA was purified using the Qiagen DNeasy Extraction kit and 0.5 µg was subjected to bisulfite treatment (Qiagen, EpiTect plus). Bisulfite-treated DNA from all tumor samples was then processed for methylation profiling using the Illumina Infinium HumanMethylationEPIC (EPIC) array (Illumina, San Diego, CA, USA) at PMGC.

### Methylation data processing

We used the open-source programming language R with software version 3.4.1. Raw IDAT data files were processed using the *minfi* Bioconductor package (version 3.3) [4] and normalized using ssNoob [13] method in the *minfi* package, which allows integration of EPIC and 450 K datasets on two different platforms. Methylation values were then measured using β values that describe the methylation levels of each CpG site (0 for unmethylated while 1 for fully methylated). We performed full quality control on all samples and removed low-quality samples with detection *p* value (detP) > 0.01. We also excluded failed probes in one or more samples with detP > 0.05. Array probes that overlapped with single-nucleotide polymorphisms (SNPs) at CpG sites (used *dropLociWithSnps* function in *minfi* package), mapped to sex chromosomes X and Y, cross reactive probes [8], or Illumina control probes were removed for unsupervised clustering. We performed batch correction prior to further exploratory analysis. Subgroups were identified using ConsensusClusterPlus Bioconductor package [39] and Silhouette score was used to identify optimal number of clusters. We performed supervised analysis of methylation data using *limma* based modeling approach (Bioconductor). Absolute mean beta value difference > 0.1 and adjust *p* value (FDR, *q* value) < 0.05 were considered to be significant. We performed unsupervised hierarchical clustering and plotted heatmaps with dendrograms (one step approach) based on the most variably methylated CpG sites using Spearman method and Ward linkage. We used Rtsne package in Bioconductor to generate tSNE plots based on the top 10,000 most variably methylated CpG sites based on MAD (median absolute deviation).

### Compound CNV plots

The "copy number" package in bioconductor was used to generate compound CNV plots based on segment information generated from DNA methylation and whole exome sequencing data. To generate compound CNV plots using the DNA methylation data, we used the “conumee” package in Bioconductor where each segment has fixed exact starting and ending point.

### Whole exome sequencing

Libraries were constructed from > 200 ng starting genomic DNA using the Agilent SureSelect Human All Exon V5 + UTRs kit. One hundred base pair paired-end reads were sequenced using Illumina HiSeq 2500 instruments at OICR-TGL (Toronto, Canada) to maximum 250 × target read depth for tumor and 50 × for matched normal tissue (blood) DNA libraries. Sequence reads were aligned against human genome reference build GrCh37 (hg19). Quality control metrics were captured within our quality control database, Shiny TGLQC. Haplotype Caller [34], MuTect1 v1.1.7 [9] and Strelka v1.0.13 [37] were run to create raw variant call files (VCFs). Raw VCF files were annotated with Variant Effect Predictor v92 [29]. Somatic variants were annotated with GnomAD r2.0.1 [25] to remove common variants. Variants were filtered against GnomAD < 0.001 (0.1%), VAF > 10% and a TGL frequency database of variants (< 10%). Variants were also annotated against known cancer hotspots v2 (CancerHotspots.org) both at the variant level and gene level. Analysis included actionable/oncogenic driver analysis using the Precision Oncology Knowledge Base (OncoKB) and pathogenic database ClinVar [7, 24]. Additional analysis was applied to detect allele specific copy number profiles, loss of heterozygosity, and to estimate ploidy/cellularity using Sequenza for matched tumor/normal pairs [12]. Mutation burden was calculated as the number of non-synonymous mutations per callable megabase. MuTect v1.1.7 [9] wig coverage file was used to determine callability.

### Whole genome sequencing

Genomic DNA (0.5–1 µg) libraries were generated using the Illumina TruSeq PCR-free DNA library preparation kit, followed by 150-base, paired-end sequencing on two lanes (60 ×) for tumors and one lane (30 ×) for matched normal samples on the Illumina HiSeqX. WGS data were aligned against hg19 using BwaMem v0.7.12 [26]. Somatic mutations were called using Mutect v1.1.7 [9] and Strelka v1.0.13 [37]. Variants with allele fractions < 5% were removed. We annotated variants using Variant Effect Predictor v 92.0 [29], OncoKB Precision Oncology Knowledge Base, CancerHotspots.org and dbNSFP database. Likely germline variants with GnomAD population frequency > 0.01% in any population (r2.0.1) were removed to retain putative somatic mutations. Allele-specific copy number profiles, loss of heterozygosity, and estimates of purity and ploidy were analyzed using Sequenza v 2.1.2 [12]. CNVs with log_2_*R* > 0.7 (high level gain) and < − 0.7 (deep deletions) were taken into account. Tumor mutation burden was calculated as the fraction of total number of protein altering somatic mutations across the entire exome space (37.2855 Mb).

### Structural variant analysis

Structural variant (SV) prediction for tumor and matched normal pairs was carried out using Delly (version 0.8.1) [35]. Output calls were filtered according to the “PASS” filter and regions such centromeres and telomeres were excluded, with the list provided with Delly developers. SVs were validated and visualized using MAVIS (version 2.2.6) [36]. SV calls were annotated with gene, transcript, and putative fusion products.

### RNA sequencing

Tumor RNA libraries were prepared from 200 ng of RNA and the Illumina TruSeq mStranded Total RNA (*N* = 18, RIN > 8) and Ribo-Zero Gold (*N* = 6, RIN < 8) kits. Libraries were pair-end sequenced for 100 cycles using the Illumina HiSeq 2000 to achieve a minimum of 80 million reads per sample. We used FusionCatcher [10] to detect novel gene fusions. To validate fusions, purified RNAs were reverse-transcribed using SuperScript VILO kit. PCR was performed on the cDNA as previously described [2]. PCR products were purified using the Qiagen MinElute PCR purification kit and run on a 1.2% agarose gel. The cDNA of *SH3PXD2A-HTRA1* fusion cloned into a Gateway compatible vector [2] served as positive control.

The quality assessment of the raw reads was carried out using the FastQC tool (version 0.11.5). The reads were aligned to the human reference genome, hg38 using the star aligner (version 2.4.2a). The RNASeq reads were counted over gene exons using HtSeq (version 0.11.0). Genes were annotated as per the Gencode Version 33 annotation file (https://www.gencodegenes.org/human/release_33.html). DEseq2 (DESeq2_1.26.0) was used to normalize and difference in library preparation methods was corrected by limma (version limma_3.42.2). Differential gene expression analysis was performed using the R package “edgeR” in BioConductor. The standard method in the EdgeR software, Quasi-likelihood *F* test, was used for DEG determination in edgeR. Pathways analysis was performed using DEG from indicated pairwise analysis by the Gene Set Enrichment Analysis (GSEA) software from the Broad Institute (https://software.broadinstitute.org/GSEA) (version 3.0). DEG results were used to calculate C6 ranking scores for each gene by *p* values and fold-changes from the analysis using the following formula:

$${\text{sign}}\left( {\log {\text{FC}}} \right)x - \log 10(p{\text{-value}}),$$

where: sign (logFC) determines the direction of the change with + ve as upregulation and –ve as down. − log10 (*p* value) determines the scale of ranking; the lower the *p* value, the higher is the score. We use this ranking score as input of the GSEA analysis.

Human_GOBP_AllPathways_no_GO_iea_June_20_2019_symbol. gmt from [https://baderlab.org/GeneSets] was used to identify enriched cellular pathways in GSEA analysis. Highly related pathways were grouped into a theme and labeled by AutoAnnotate (version 1.2) in Cytoscape (Version 3.7.2). GSEA results were visualized using the Enrichment Map app (Version 3.1) in Cytoscape.

We used CIBERSORT Bioconductor package to perform deconvolution and estimate the abundances of cell types (quantify immune cell proportions) in our RNAseq dataset.

### Germline mutations scanning and somatic mutations detection in tumors

Library preparation for NGS was accomplished using the HaloPlex PCR target enrichment system (Agilent Technologies Inc.). Using SureDesign (Agilent Technologies Inc.), probes were generated to cover the exons and the UTR regions of the following genes: *NF2*, *SMARCB1,* and *LZTR1* (NCBI Nucleotide database, https://www.ncbi.nlm.nih.gov/nucleotide; Accession numbers NM181832.2, NM003073.3, NM006767.3). Sequencing was performed using MiSeq reagent kit version 2, 300 cycles, on the MiSeq instrument. Amplicon reads were aligned against the human reference genome hg19 with BWA MEM.

For the sporadic schwannomas (NS-SWN), all patients have a screening genetic testing done in the NF clinic and there were no positive germline *LZTR1, SMARCB1,* and NF2 cases among the samples used in this study.

### *NF2* somatic mutations detection

The entire coding sequence of *NF2* was sequenced with PCR and capillary sequencing on Biosystems 3100 or 310 Capillary DNA Analyzer. Primer sequences are available on request.

### Microsatellite analysis

Loss of heterozygosity (LOH) in 22q was investigated using microsatellites D22S420, D22S539, D22S1174, D22S315, D22S1154, D22S1163, D22S280, D22S277, D22S283, D22S423, D22S274, and D22S1169 from the ABI PRISM Linkage Mapping set version 2.5 (Applied Biosystems).

### Multiplex ligation probe amplification

Copy number changes (deletions or duplications) of 22q loci were validated by Multiplex Ligation-Dependent Probe Amplification (MLPA) when fresh tumor tissues were available. *SMARCB1, NF2* and 22q11 MLPA test kits (MRC-Holland, P044_B1, P258_C1 and P324_A2) were used and electrophoresis data were analyzed using GeneMapper software (Life Technologies).

### Oncoprints

We generated oncoprints using the R package complex heat maps50. Frequencies of events were adjusted to the number of samples that could be annotated for the respective event (that is, samples where we could not call CNVs were not counted and shaded light gray for CNV relevant genes). Subgroup enrichment for specific genes was determined using Fisher’s exact test and a threshold of the Benjamini–Hochberg-adjusted *p* value (*p* ≤ 0.05).

### Statistics

Chi-square statistics were used to compare binomial variables between groups. Spearman coefficients were used for comparisons of continuous variables. For direct comparisons, an unpaired two-tailed Student’s *t* test was used. Missing data were omitted from analyses.

## Results

### Cohort summary

Our cohort included 165 SWNs from 72 SWNTS patients (female/male: 31/41). The samples were analyzed for DNA methylation signatures, coding mutations, copy number variations (CNVs), structural variations (SVs), transcriptional profile, and the presence of gene fusions. For comparison, we performed DNA methylation profiling on 90 neurofibromas, including 56 plexiform and 34 cutaneous tumors (Online Resource Table 1).

### DNA methylation signature of SWNTS-SWNs

**Fig. 1:**
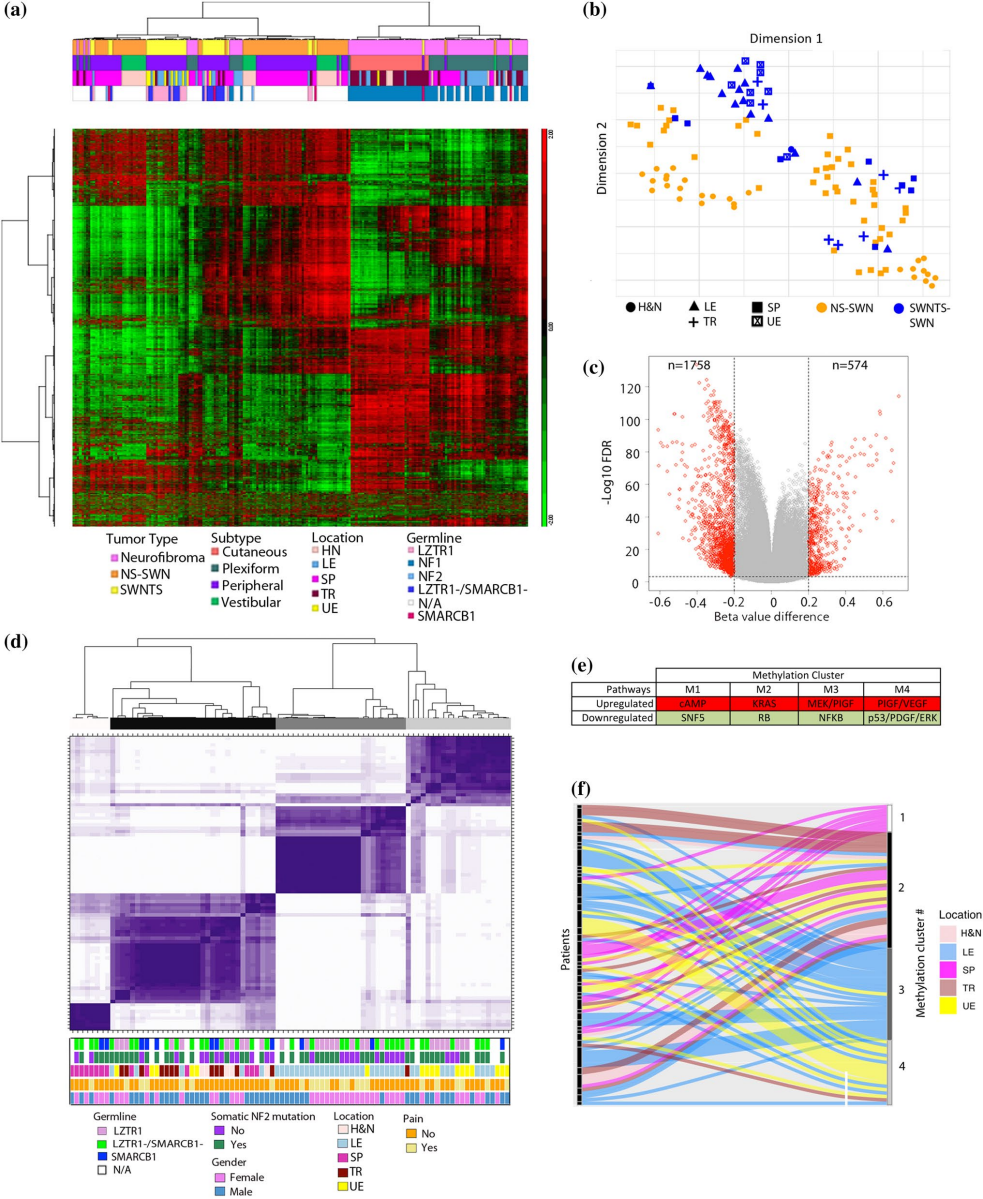
DNA methylation landscape of SWNTS-SWNs in the spectrum of benign PNSTs. **a** Unsupervised hierarchical clustering of the top 2000 most variably methylated CpG sites in SWNTS-SWNs (*N* = 42), NS-SWNs (*N* = 73), NF2-SWNs (*N* = 2), and NF (*N* = 90). Color codes to the right denote features including tumor type, anatomic location, and germline mutations. **b** tSNE plot generated using the top 10,000 most variably methylated CpG sites in SWNTS-SWNs and NS-SWNs. Symbols denote anatomic location associated with each tumor. **c** Supervised analysis shows differentially methylated CpG sites between SWNTS-SWNs and NS-SWNs (absolute mean beta value difference > 0.2, *q* < 0.0005) presented as a volcano plot. **d** Consensus clustering of the 2000 most variably methylated CpG sites in SWNTS-SWNs (*N* = 88) showing four stable clusters. Color codes to the right denote germline mutations status, tumor location, somatic mutations in *NF2*, sex, and pain reported by the patients. **e** Key pathways differentially regulated in each methylation cluster based on GSEA C6 analysis. **f** Sankey plot showing the distribution of tumors from different anatomic locations resected from same individuals across the four methylation clusters. Tumor location codes: *LE* lower extremity, *UE* upper extremity, *SP* spinal, *TR* truncal, *H&N* head and neck

The majority of peripheral nerve sheath tumors (PNSTs) are derived from the Schwann cell lineage and comprise diverse histological subgroups [2, 31]; however, no comprehensive studies to date have examined the DNA methylation landscape of SWNTS-SWNs in comparison to neurofibromas and the histologically indistinguishable NS-SWNs. Our DNA methylome profiling demonstrated a robust separation between SWNs and neurofibromas (Fig. 1a). In contrast, there was no clear separation between SWNTS-SWNs and NS-SWNs, suggesting that they arise from the same cell of origin. Further, we found no differences in the DNA methylation profile of SWNTS-SWNs harboring germline mutations in *LZTR1* or *SMARCB1* (Online Resource Fig. 1a). However, SWNTS-SWNs from extremities (arms and legs) were separated from other anatomic locations (Fig. 1b, Online Resource Fig. 1b).

Comparison of SWNTS-SWNs with NS-SWNs revealed no distinction among these tumors upon unsupervised hierarchical clustering (Online Resource Fig. 1c) but continued to show that SWNTS-SWNs from extremities separated from all other anatomic locations. Additionally, supervised comparison of differentially methylated CpGs revealed that 75% of these CpGs were hypomethylated in SWNTS-SWNs relative to NS-SWNs (Fig. 1c), the majority of which were located within CpG islands and in promoter regions (Online Resource Fig. 1d, Online Resource Table 2). To validate this, as a surrogate measure of global DNA methylation status, we assessed the DNA methylation level of repetitive elements *ALU* and long interspersed elements-1 (*LINE-1*). *LINE-1* elements were significantly hypomethylated in SWNTS-SWNs relative to NS-SWNs (Student’s *t* test, *p* = 0.0005, Online Resource Fig. 1e), concomitant with reduced methylation in the promoter region and higher transcription of *L1TD1* gene (encoded within *LINE-1* elements) in SWNTS-SWNs relative to NS-SWNs (fold change = 5.17, *q* = 0.039). These results were corroborated by higher expression of ten-eleven translocation 1 and 2 (*TET1* and *TET2*) genes, which promote DNA demethylation (Online Resource Fig. 1f).

Analysis of the DNA methylation profile of germline *LZTR1*-mutant in comparison to *LZTR1-*wild-type SWNTS-SWNs demonstrated that the majority of differentially methylated CpGs were hypomethylated (Online Resource Fig. 1g); however, no changes were detected in the methylation status of *LINE-1* or *ALU* elements or the expression of *L1TD1*, *TET1*, *TET2*, *DNMT1*, or *DNMT3A* genes (data not shown), suggesting that *LZTR1*-mutant samples are not globally hypomethylated relative to *LZTR1* wild-type. Consensus clustering (Fig. 1d) and unsupervised hierarchical clustering (Online Resource Fig. 1h) of SWNTS-SWNs alone generated four stable clusters, which were specifically associated with the anatomic location of tumors. We found deregulations in prominent transcriptional programs associated with each methylation cluster including upregulation of cAMP, KRAS, MEK/PIGF, and PIGF/VEGF pathways in clusters 1–4, respectively (Fig. 1e). Moreover, multiple tumors resected from different anatomic regions of the same individuals resolved into different clusters (Fig. 1f), further indicating that there are distinct DNA methylation signatures associated with Schwann cells of origin from different regions of the body.

### Spectrum of somatic alterations in SWNTS-SWNs

**Fig. 2:**
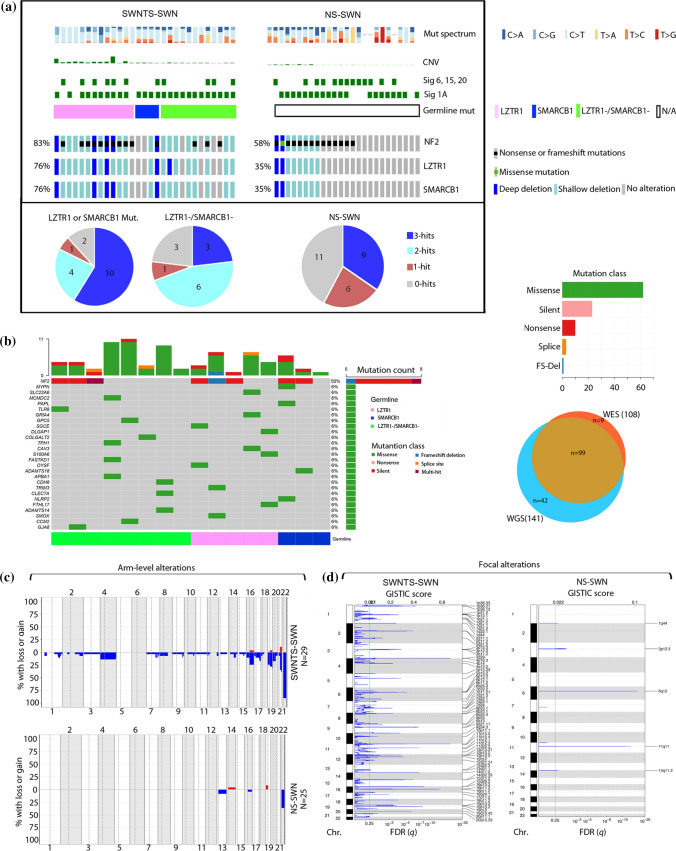
Landscape of somatic alterations in SWNTS-SWNs. **a** Oncoprint showing results from WES analysis of 29 SWNTS-SWNs and 25 NS-SWNs. Mutation spectrum and CNV fraction are plotted on the top. COSMIC signatures 1A, 6, 15, and 20 in each cohort are also depicted. Somatic mutations and deletions in *NF2*, *LZTR1*, and *SMARCB1* are shown. Distribution of zero, one, two, or three somatic hits across the two cohorts are shown as pie charts below. **b** Oncoprint showing top deleterious somatic variants (based on SIFT and PolyPhen2) identified by both WES and WGS methods in 16 overlapping samples. Clinical and molecular annotations are depicted above and below the oncoprint. Venn diagram to the right shows the number of variants called by both WES and WGS. **c** Plots showing compounded arm-level CNV in SWNTS-SWNs versus NS-SWNs. **d** GISTIC plots showing significant focal deletions in SWNTS-SWNs and NS-SWNs

Very little has been examined in PNSTs, and specifically in SWNTS-SWNs, with respect to somatic single nucleotide variants (SNVs), copy number variations (CNVs), and structural variations (SVs). Here, we performed WES (*N* = 29) and WGS (*N* = 22) on SWNTS-SWNs with matched normal DNA from blood. We demonstrated that majority of somatic mutations were C > T transitions (Fig. 2a) and concordantly revealed that four signatures from the catalog of somatic mutations in cancer (COSMIC) database were predominantly present in at least two SWNTS-SWNs and contributed to at least 5% of the mutations (Fig. 2a, Online Resource Table 3). Signature 1A (MUTYH) was the most prominent (26/29), followed by mismatch repair (MMR)-related signatures 6 (7/29) and 15 (4/29), and signature 2 (APOBEC) (3/29). These signatures were similarly operative in NS-SWNs (Online Resource Table 3) except for signature 2, which was absent in NS-SWN cases, whereas signature 20 was present in 3/24 NS-SWN cases and only in one SWNTS-SWN.

With respect to tumor mutation burden (TMB), both SWNTS-SWNs and NS-SWNs demonstrate an overall low TMB in comparison with most other tumor types (Online Resource Fig. 2a). SWNTS-SWNs (*n* = 28) and NS-SWNs (*N* = 25) displayed a similar TMB: SWNTS-SWNs (WES = 0.17/Mb or 5.92/sample, WGS = 0.23/Mb or 8.81/sample) and NS-SWNs (WES = 0.22/Mb or 6.08/sample). Furthermore, although we found COSMIC signatures associated with MMR-related mutations, we did not detect instability in 22q microsatellites, suggesting that MMR signatures are not attributed to hypermutation or microsatellite instability in SWNTS-SWNs. *NF2* gene was the only recurrent driver somatic mutation in all SWNs with interestingly a statistically significant higher proportion of SWNTS-SWNs harboring mutations and copy number loss in *NF2* compared to NS-SWNs (83% vs. 58%, Chi-square, *p* < 0.0001). Notably, all SWNTS-SWNs with germline mutations in *LZTR1* harbored either mutations or deletions in *NF2* (Fig. 2a).

Additional novel deleterious somatic single nucleotide variants (SNVs) were detected in SWNTS-SWNs by both WES and WGS and validated by Sanger sequencing in several genes including *MYPN*, *CAV3*, and *SMOX* (Fig. 2b)*.* However, these alterations were not recurrent, suggesting that a set of low-frequency alterations may play an unappreciated, yet important role in the pathogenesis of SWNTS-SWNs (Online Resource Table 4). In addition, all point mutations and variant allele frequencies that we identified in *NF2, LZTR1,* and *SMARCB1* were similar to those reported previously [40] (Online Resource Fig. 2b).

### Structural aberrations in SWNTS-SWNs

To date, recurrent chromosomal deletions in SWNs have only been reported in chromosome 22q. Our WES analysis revealed that majority of chromosomal arm-level copy number events corresponded to deletions in chromosome 22q (22q11–q13) (Fig. 2a, c), and a statistically significantly higher proportion of SWNTS-SWNs harbored deletions in this region compared to NS-SWNs (80% vs. 30%, Chi-square, *p* < 0.01) (Fig. 2c). These results were supported by copy number analysis using WGS (Online Resource Fig. 2c) and DNA methylation data (Online Resource Fig. 2d) and were in agreement with an overall higher fraction of genome altered (CNV) in SWNTS-SWNs (WES = 5.97%, WGS = 2.40%) compared with NS-SWNs (WES = 0.86%, Student’s *t* test, *p* = 0.005).

Our copy number analysis indicated that both *LZTR1* and *SMARCB1* genes, located on chromosome 22q along with *NF2*, were recurrently deleted in both SWNTS-SWNs and NS-SWNs, with a statistically significant higher proportion of SWNTS-SWNs showing deep deletions in all three genes (76% vs. 35%, Chi-square, *p* < 0.01, Fig. 2a). We found a large number of other genes that were recurrently deleted on 22q and the expression of majority of these genes were significantly lower in SWNTS-SWNs relative to NS-SWNs (*p* < 0.01, Online Resource Fig. 2e). These findings emphasize the potential role of genes on 22q—other than *LZTR1*, *SMARCB1,* and *NF2*—in the pathogenesis of SWNTS-SWNs. The genes of significance include *MAPK1, BCR, EWSR1, PATZ1, ZNRF3, *and* MYH9,* which were among the most frequently deleted (77%) and are situated proximal to *SMARCB1* (22q11.21–q11.23).

In addition to 22q deletions, we detected recurrent arm-level deletions in chromosomes 4, 16, 19, and 21 (Fig. 2c), and interestingly only in *LZTR1-*mutant samples (Online Resource Fig. 2f). These results were consistent with ninefold higher CNV in *LZTR1*-mutant compared to *LZTR1-*wild-type SWNTS-SWNs (Student’s *t* test, *p* = 0.0072). We also discovered several focal non-22q deletions across the genome in SWNTS-SWNs that were absent in NS-SWNs (Fig. 2d).

It is thought that SWNTS-SWNs arise as a result of a three-step/four-hit mutational event [22]: one germline mutation in *SMARCB1* or *LZTR1,* followed by one somatic mutation in *NF2,* and then two additional somatic alterations upon co-deletion of these genes on 22q. We divided SWNTS-SWNs into four groups depending on the number of classical somatic hits based on WES: three hits (22q deletion and *NF2* mutation), two hits (22q deletion), one hit (*NF2* mutation), or zero hits (neither). Of note, nine (31%) SWNTS-SWN samples displayed two hits (without somatic mutations in *NF2*) [33], while this was not observed in NS-SWNs (Fig. 2a), once again suggesting that a large number of other genes located on 22q contribute to the pathogenesis of SWNTS-SWNs.

**Fig. 3:**
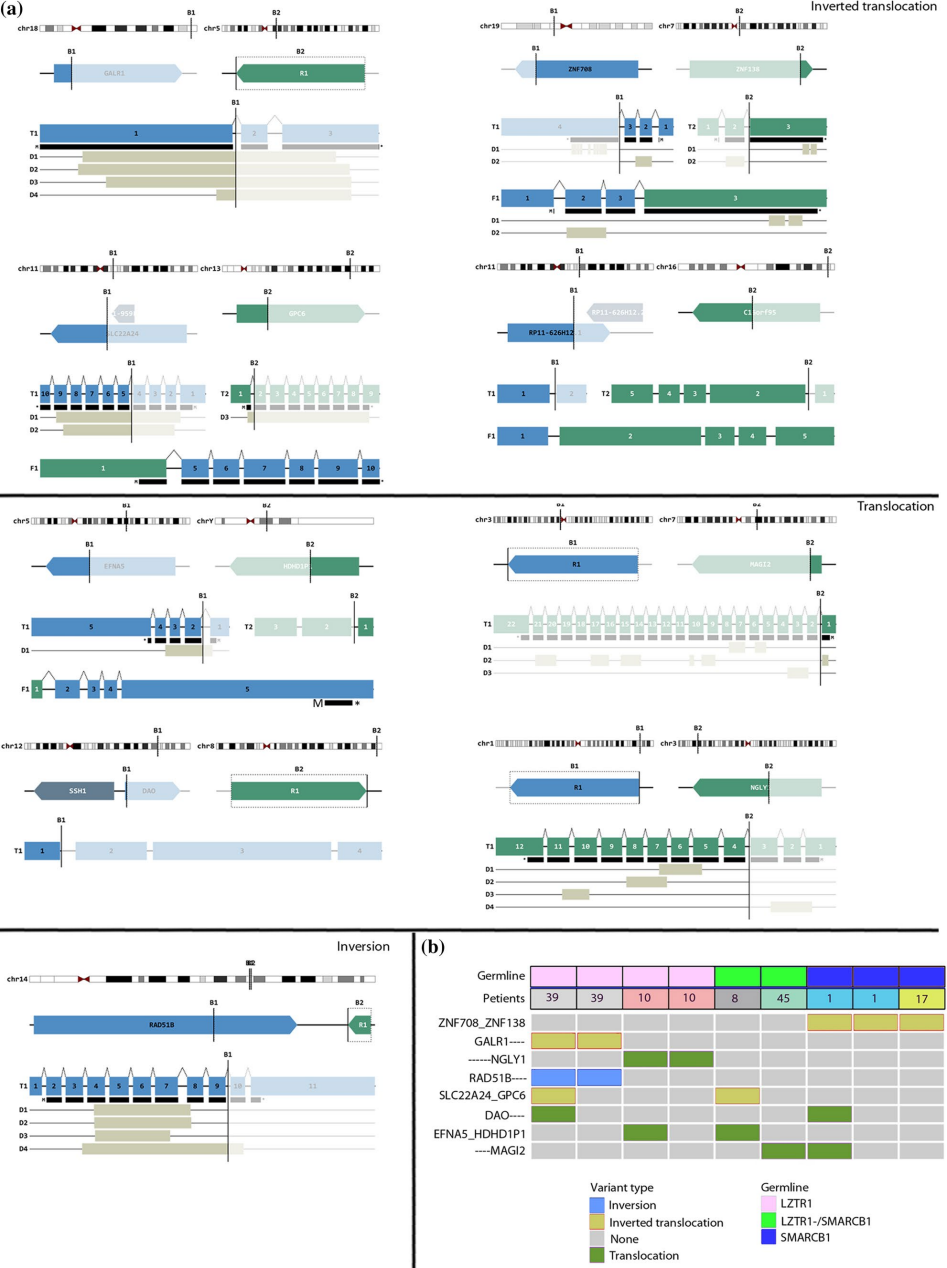
Structural variants in SWNTS-SWNs. **a** Diagrams showing the SVs identified in SWNTS-SWNs using WGS data on 22 tumors. Only recurrent SVs present in at least 2 samples are shown. The breakpoints are given by B1 and B2. The top level depicts the breakpoint positions in each chromosome, the second level down shows breakpoints within the related genes, and the third level shows the transcripts. The predicted fusion product is shown at the bottom. Domains (D) are shown below the transcript and the bar immediately underneath each transcript (labeled with M and *) represents the open reading frame. **b** Oncoprint shows selected recurrent SVs in at least two samples. Color codes denote germline mutation status and SV type

We then assessed SVs in SWNTS-SWNs within the coding space using WGS data. We found one recurrent inversion, four inter-chromosomal translocations, and four inter-chromosomal inverted translocations in at least 2/22 tumors (Fig. 3a, Online Resource Table 5). The majority of the cases harboring these alterations were also positive for germline mutations in *SMARCB1* or *LZTR1*. The most frequent (3/22) alteration was an inverted translocation between two zinc finger proteins: ZNF708 on Chr. 19p and ZNF138 on Chr. 7q. However, the significance of this rearrangement is not known. Furthermore, examination of different tumors from same individuals showed that not all tumors had the same profile, with some tumors harboring more SVs compared to the other tumors from the same individual (Fig. 3b).

### Transcriptome profile of SWNTS-SWNs

**Fig. 4:**
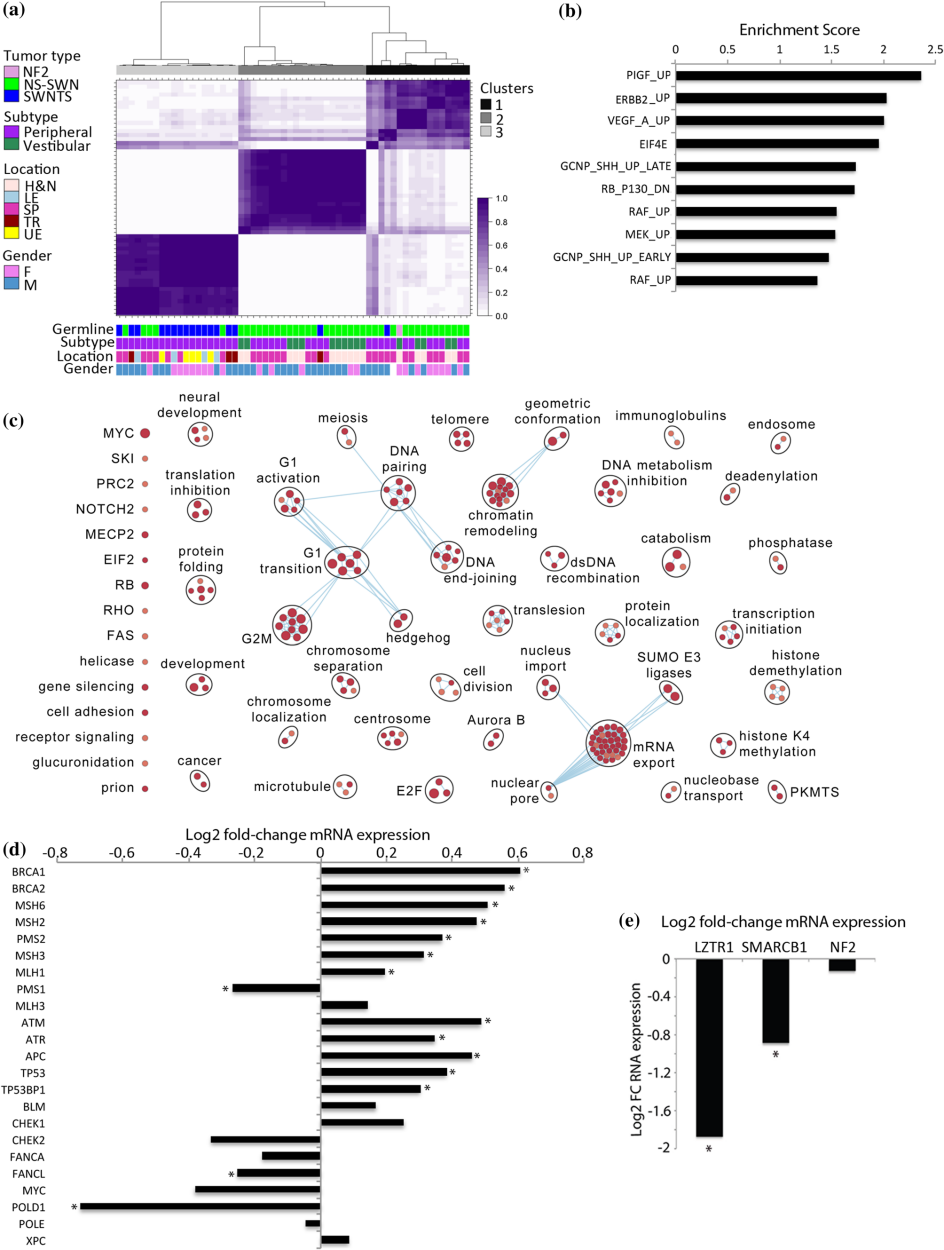
Transcriptome hallmarks of SWNTS-SWNs. **a** Consensus clustering of top 2500 differentially expressed genes in SWNTS (*N* = 18) and NS-SWNs (*N* = 41) using Spearman correlation and Ward linkage. Annotations are depicted to the left and below the diagram. **b** Plot showing the top ten highest enrichment scoring pathways (*p* < 0.0001) based on GSEA C6 analysis of DEGs in SWNTS versus NS-SWNs. **c** Analysis of top differentially regulated pathways in SWNTS-SWNs (*N* = 24) versus NS-SWNs (*N* = 41). Upregulated pathways are labeled in red. Pathways with high degree of gene overlap are connected with blue lines. **d** Fold-change expression of DDR related genes in SWNTS-SWN versus NS-SWN (* denotes *p* < 0.01). **e** Plot showing log2 fold-change in expression of LZTR1, SMARCB1, and NF2 in SWNTS-SWNs (*N* = 24) versus NS-SWNs (*N* = 41)

We compared the gene expression profiles of SWNTS-SWNs with NS-SWNs and performed consensus clustering of top differentially expressed genes (Online Resource Table 6), demonstrating that SWNTS-SWNs (*N* = 18) partitioned mainly into one cluster, while NS-SWNs generated two separate clusters (Fig. 4a), suggesting that SWNs that arise in SWNTS patients harbor a distinct transcriptome profile. Further, gene set enrichment analysis (GSEA) of key oncogenic pathways indicated that the majority of upregulated pathways in SWNTS-SWNs relative to NS-SWNs included PIGF, VEGF, MEK, ERBB2, and SHH, while RB was the highest scoring downregulated pathway (Fig. 4b). We further depicted the deregulated genes and associated pathways, demonstrating that cell division, cell cycle, and DNA repair related mechanisms were upregulated in SWNTS-SWNs relative to NS-SWNs (Fig. 4c). Since SWNTS-SWNs harbored higher CNV and 22q LOH relative to NS-SWNs and both tumor types displayed the MMR COSMIC signatures 6 and 15, we assessed the expression of MMR and DNA repair-related genes and found that *MSH3*, *MSH6*, *PMS2* and *MLH3* were significantly upregulated in SWNTS-SWNs relative to NS-SWNs (Student’s *t* test, *q* < 0.01, Fig. 4d). While recurrent mutations were absent in any of these genes, *MSH6* and *MLH1* were significantly hypomethylated in SWNTS-SWNs relative to NS-SWNs, suggesting that DNA demethylation might be one mechanism that led to relatively higher expression of these genes (data not shown). Moreover, a statistically significant lower expression of *LZTR1* and *SMARCB1* genes was detected in SWNTS-SWNs, which was consistent with more extensive deletion of these genes in these tumors compared with NS-SWNs (*q* < 0.001, Fig. 4e).

Previous reports have comprehensively delineated the role of LZTR1 in inhibiting the RAS/MAPK pathway activation by promoting the ubiquitination and degradation of RAS [5]. Mutations in *NF2* are also known to activate RAS signaling, in part through the Hippo pathway [14]. We found that the RAS/MAPK pathway was indeed upregulated in germline *LZTR1*-mutant versus *LZTR1-*wild-type and somatic *NF2*-mutant versus *NF2*-wild-type SWNTS-SWNs (Online Resource Fig. 3a), suggesting that these mutations likely sensitize SWNs to MEK inhibitors. We further assessed the top deregulated pathways in germline *LZTR1*-mutant versus *LZTR1*-wild-type, germline *SMARCB1-*mutant versus *SMARCB1-*wild-type, and somatic *NF2*-mutant versus *NF2-*wild-type SWNTS-SWNs (Online Resource Fig. 3b). Germline *LZTR1* mutations resulted the largest number of deregulated pathways, in particular, downregulation of several inflammatory and immune-related pathways, among others.

We have previously reported and characterized a novel gene fusion between *SH3PXD2A* and *HTRA1* genes in approximately 10% of NS-SWNs [2]. Using RT-PCR we detected this fusion in SWNTS-SWNs (14%), consistently with a male predominance (14/22, 63%). Interestingly, majority of fusion-positive samples harbored germline mutations in *LZTR1* (15/22, 68%, Chi-square, *p* = 0.025). Similar to NS-SWN presence of this fusion has direct therapeutic application with use of MEK inhibitors [2]. Further analysis of the transcriptome data identified several other fusions seen at a significantly higher frequency in SWNTS-SWNs compared to NS-SWNs (Online Resource Table 7) including *NAIP1*-*OCLN* on chromosome 5 [15] (67% in SWNTS-SWNs vs. 29% in NS-SWNs, Chi-square, *p* < 0.01). Another notable fusion is the previously reported cancer predisposition *KANSARL* fusion between *KANSL1* and *ARL17A* genes on chromosome 17q21.31; however, in similar proportions in SWNTS-SWNs and NS-SWNs (Online Resource Fig. 4) [45].

### The immune gene signature of SWNTS-SWNs

**Fig. 5:**
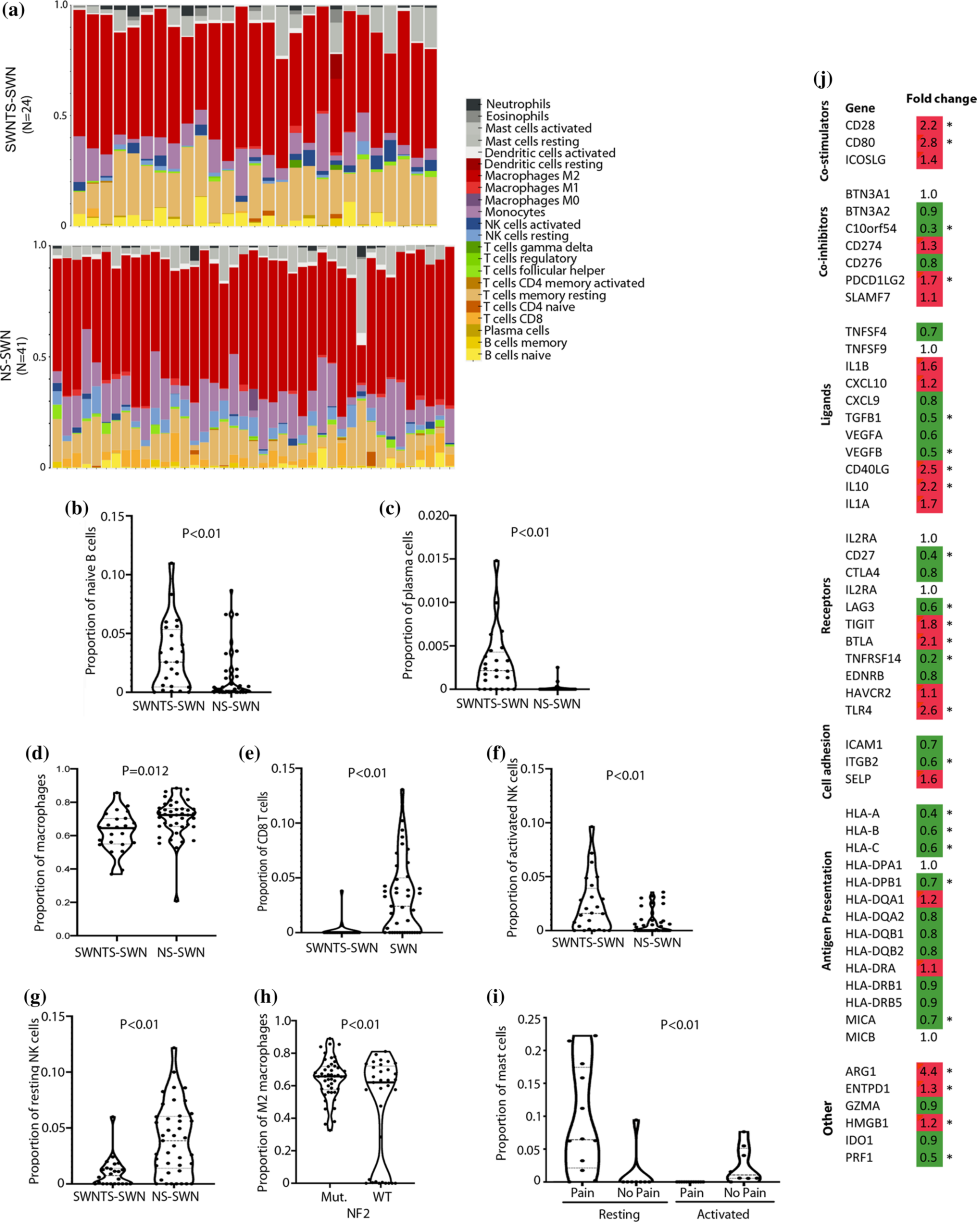
Immune gene signature of SWNTS-SWNs. **a** Estimated proportion of different immune cell types in SWNTS-SWNs and NS-SWNs based on RNAseq data. **b** Plots showing significantly different proportions of naïve B cells, **c** plasma cells, **d** macrophages, **e** CD8^+^ T cells, **f** activated NK, and **g** resting NK cells in SWNTS-SWNs versus NS-SWNs. **h** Plot showing proportion of M2 macrophages in *NF2*-mutant (*N* = 16) versus *NF2*-wild-type (*N* = 7) SWNTS-SWNs. **i** Plot showing proportion of resting and activated mast cells in painful (*N* = 12) versus non-painful (*N* = 8) SWNTS-SWNs. **j** Analysis of mRNA expression of immune regulatory genes in SWNTS-SWNs (*N* = 24) versus NS-SWNs (*N* = 41)

Several reports have pointed to the importance of the nerve microenvironment in development of SWNs. In particular, the role of M2-polarized macrophages, with anti-inflammatory but pro-tumorigenic functions, in *NF2*-deficient SWNs has been demonstrated in NF2 mouse models [38]. We evaluated the proportion of different immune cells infiltrating SWNs using our RNAseq and demonstrated that SWNTS-SWNs displayed a statistically higher proportion of naïve B cells, plasma cells, and activated natural killer (NK) cells, but lower number of total macrophages and CD8^+^ T cells compared to NS-SWNs (Student’s *t* test, *p* < 0.05, Fig. 5a–g). We found that *NF2*-mutant SWNTS-SWNs had a statistically greater proportion of M2 macrophages compared to *NF2*-wild-type samples (Student’s *t* test, *p* < 0.01, Fig. 5h). In addition, the expression of the majority of key immune regulatory genes (HLA-A, -B, -C, and -DPB1) were decreased, while genes involved in T cell stimulation (CD28 and CD80) were mainly upregulated in SWNTS-SWNs relative to NS-SWNs (Student’s *t* test, *p* < 0.05, Fig. 5j).

### Genomic hallmarks of pain in SWNTS-SWNs

While some SWNs that arise in SWNTS are characterized by extensive noxious pain, others are pain free in the same individual. However, the biological alterations underlying pain in SWNTS-SWNs are poorly understood. We found a number of key correlates to localized pain. A statistically significant proportion of painful SWNTS-SWNs were from the lower extremities (LE, 58%, Chi-square, *p* = 0.001) (Online Resource Fig. 5a), occurred predominately in female patients (64%, *z* score, *p* < 0.001), patients with germline mutations in *LZTR1* (57%, Chi-square, *p* = 0.002), and also tumors that harbored the *SH3PXD2A-HTRA1* gene fusion (16%, *z* score, *p* = 0.039). WES results indicated that painful SWNTS-SWNs had 4.4-fold higher CNV (*p* = 0.042), while TMB was not significantly different in painful SWNTS-SWNs. Notably, painful SWNTS-SWNs displayed significant upregulation of the RAS/MAPK pathway (Online Resource Fig. 5a), and activation of this pathway is thought linked with pain. We found significant upregulation of other key oncogenic pathways including PIGF/VEGF, ERBB2 (HER2), RB/RAF, mTOR, and MEK, among others, in painful SWNTS-SWNs (Online Resource Fig. 5c). These pathways were also significantly upregulated in tumors from extremities, validating further the association of pain with tumor location. Of these, ERBB2/HER2 and VEGF pathways were significantly upregulated is *LZTR1*-mutant tumors. Furthermore, several pain-related genes including *MMP16, GABRB3, NRP1, MMP1,* and *TGFBR2* were significantly upregulated in painful SWNTS-SWNs (Online Resource Fig. 5d). Transcriptional assessment of immune cell infiltrates revealed that painful SWNTS-SWNs consisted of a statistically higher proportion of total mast cells (Student’s *t *test, *p* < 0.01, Fig. 5i) that are well recognized in modulating nociceptive pain [17].

## Discussion

**Fig. 6:**
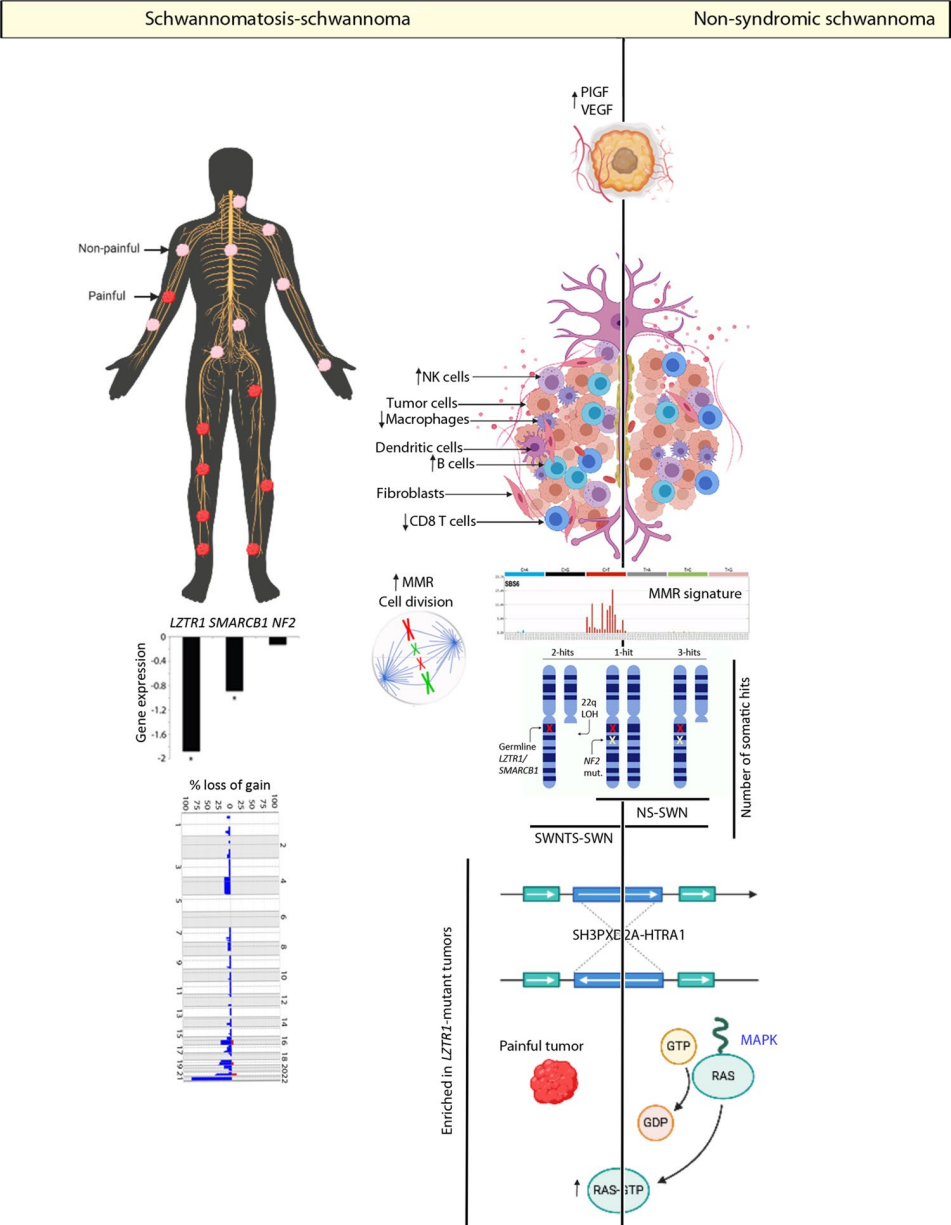
Summary of key molecular alterations in SWNTS-SWNs. Diagram showing key genomic alterations detected in SWNTS-SWNs and in comparison with NS-SWNs. These include differences in immune cell proportions, CNV, somatic alterations in chromosome 22q, and anatomic location associated with pain in SWNTS-SWNs. We also depicted similarities among SWNTS-SWNs and NS-SWNs with respect to COSMIC MMR signatures, presence of *SH3PXD2A-HTRA1* gene fusion, and activation of key oncogenic pathways

Identification of the molecular drivers of genetic cancer predisposing conditions has led to considerable advance in understanding the pathophysiology of tumor formation. Although histologically identical to NS-SWNs, based on their clinical presentation and what is known of their genetic alternations, evidence points to SWNTS-SWNs harboring a very distinct phenogenomic profile. To explore this, we conducted the first comprehensive genomic analysis of SWNTS-SWNs and performed detailed comparison with NS-SWNs (Fig. 6), leveraging the largest cohort of SWNTS-SWNs.

Here, we established that the global DNA methylation signatures of SWNTS-SWNs and NS-SWNs are distinct from neurofibromas. Further, while SWNTS-SWNs did not resolve from NS-SWNs based on DNA methylation signatures, they were relatively hypomethylated across the genome and within repetitive elements. Most notably, within SWNTS-SWNs, we identified four DNA methylation subgroups, associated with tumor anatomic location, hence suggesting the cells of origin are distinct based on tumor location. Furthermore, each methylation cluster was associated with distinct transcriptome profile with relative upregulation of transcriptional programs including cAMP, NFkB, RB, and PIGF, further pointing to the existence of four subtypes of SWNTS-SWNs. Thus, the methylation subclasses can influence decision making for clinical care and exploring therapeutic avenues according to the dominant expression pathway.

Results from somatic genomic analysis point to a significantly lower stability of the genome in SWNTS-SWNs compared to their sporadic counterparts. While both SWNTS-SWNs and NS-SWNs harbor low TMB, SWNTS-SWNs display significantly greater chromosomal copy number aberrations relative to NS-SWNs and significantly higher proportion of samples display chromosome 22q LOH. Here, we report recurrent novel non-22q deletions in chromosomes 4, 16, 19, and 21, specifically prevalent in *LZTR1*-mutant samples. The relatively higher CNV in SWNTS-SWNs is mainly due to gross chromosomal aberrations rather than point mutations or microsatellite instability.

We also show upregulation of distinct actionable transcriptional programs including angiogenesis-related pathways, PIGF and VEGF, in addition to SHH and MEK in SWNTS-SWNs relative to NS-SWNs. Consistent with our pathway analysis indicating activation of DNA repair and cell cycle-related pathways, we found significantly higher transcription of MMR and DNA repair-related genes, in particular *BRCA1/2* and *MSH 2/3/6*, in SWNTS-SWNs relative to NS-SWNs [44].

We previously reported on a novel fusion that has therapeutic potential in sporadic schwannomas (i.e., NS-SWNS). Here, we report the presence of the *SH3PXD2A-HTRA1* gene fusion for the first time in SWNTS-SWNs and show that its prevalence is significantly associated with germline *LZTR1* mutations and tumor-associated pain. Given the direct therapeutic significance of the fusion, consideration for use of MEK inhibitors as a therapeutic strategy for pain management in this patient population is promising.

Overall, we note a considerable association between specific molecular alterations in SWNTS-SWNs and germline mutations in *LZTR1* (Fig. 6). These include higher prevalence of somatic mutations and deletions in *NF2*, higher CNV, and prevalence of pain and the *SH3PXD2A-HTRA1* fusion. Recent studies have demonstrated a prominent role for *LZTR1* in regulating the activation of the oncogenic RAS/MAPK signaling pathway [1, 5]. Thus, activation of the DNA damage response and chromosomal instability seen here in samples with *LZTR1* mutations may be in part due to the recognized role of RAS activation [44]. Additionally, it is well documented that MAPK activation plays a role in peripheral and central nervous system sensitization to extensive noxious stimuli [20, 21]. We demonstrate upregulation of the RAS/MAPK pathway in SWNTS-SWNs that harbor mutations in *LZTR1* or *NF2*, tumors in extremities, tumors with *SH3PXD2A-HTRA1* gene fusion, and tumors associated with pain. Collectively, these key findings provide compelling evidence to exploring MAPK as a therapeutic strategy. Recent FDA approval for use of MEK inhibitors for management of tumor size and pain in NF1 disease [16] lends further hopes that targeted approaches will be of therapeutic value in SWNTS.

Several other key therapeutic avenues can be considered from the results of this study. Painful SWNTS-SWNs and tumors from extremities also show distinct upregulation of mTOR, a pathway with an established role in the initiation and maintenance of chronic pain [27]. Importantly, we also found activation of angiogenesis-regulating pathways including PIGF, VEGF, and RAF in painful tumors, suggesting that already existing drugs, such as the anti-angiogenic drug Avastin or compounds targeting PIGF, can be utilized for management of pain or tumor size in SWNTS. These compounds can be tested in clinical trials to assess alleviation of pain and improvement in patients’ quality of life as the primary end points of SWNTS treatment.

## Electronic supplementary material

Below is the link to the electronic supplementary material.

Supplementary file1 (PDF 1863 kb)
