# Supplementary material for: Epigenomic, genomic, and transcriptomic landscape of schwannomatosis
Source: Acta Neuropathol. 2020 Oct 6;141(1):101–16. doi: 10.1007/s00401-020-02230-x (PMC7785562; doi:10.1007/s00401-020-02230-x)
Supplement: Supplementary file 1 — Supplementary file1 (PDF 1863 kb) [file 401_2020_2230_MOESM1_ESM.pdf]

Online Resources

Epigenomic, genomic, and transcriptomic landscape of schwannomatosis

Sheila Mansouri[1‡], Suganth Suppiah[1‡], Yasin Mamatjan[1‡], Irene Paganini[2‡], Jeffrey C. Liu[1], Shirin Karimi[1], Vikas Patil[1], Farshad Nassiri[1], Olivia Singh[1], Yogi Sundaravadanam[3], Prisni Rath[3], Roberta Sestini[2], Francesca Gensini[2], Sameer Agnihotri[4], Jaishri Blakeley[5], Kimberly Ostrow[5], David Largaespada[6], Scott R. Plotkin[7], Anat Stemmer-Rachamimov[7], **Marcela Maria Ferrer[8]**, Trevor J. Pugh[3], Kenneth D. Aldape[9], Laura Papi[2], Gelareh Zadeh[1*]

[1] Princess Margaret Cancer Center and MacFeeters-Hamilton Center for Neuro-Oncology Research, University Health Network, Toronto, ON, Canada
[2] The Department of Experimental and Clinical Biomedical Sciences "Mario Serio", Medical Genetics Unit, University of Florence, Florence, Italy
[3] Ontario Institute for Cancer Research, Toronto, ON, Canada
[4] Children's Hospital, Department of Neurological Surgery, University of Pittsburgh, PA, USA
[5] Johns Hopkins University, Baltimore, MD, USA
[6] Department of Paediatrics, University of MN, USA
[7] Department of Pathology, Massachusetts General Hospital, Boston, MA, USA
[8] Hospital de Clínicas "José de San Martín", División de Neurocirugía and División Genética, Universidad de Buenos Aires, Buenos Aires, Argentina
[9] Laboratory of Pathology, Center for Cancer Research, National Cancer Institute, Bethesda, MD, USA

‡ These authors contributed equally to the manuscript

*** Corresponding author:**
Gelareh Zadeh, MD, PhD, FRCS(C)
Senior Scientist, Princess Margaret Research Institute
Head, Division of Neurosurgery, Toronto Western Hospital
Co-Director, Krembil Brain Institute
Wilkins Family Chair in Brain Tumor Research
14-701 PMCRT, 101 College St.,
Toronto, ON, Canada, M5G 1L7
Email: Gelareh.zadeh@uhn.ca
Tel: 416-634-8728

Online resource figure legends

Online resource figure 1. DNA methylation landscape of SWNTS-SWNs in the spectrum of benign PNSTs. (**a**) tSNE plots generated based on the top 10,000 most variably methylated CpGs in SWNTS-SWNs (N=42), NS-SWNs (N=73), NF2-SWN (N=2), and NF (N=90). Color codes denote tumor type (SWNTS-SWNs, NS-SWNs, NF2-SWN, or NF) and symbols denote tumor germline mutation status or (**b**) tumor location. (**c**) Unsupervised hierarchical clustering of the top 2,000 most variably methylated CpGs in SWNTS-SWNs and NS-SWNs (excluding NF). (**d**) Plot showing the methylated status of top variable CpGs sites in SWNTS-SWNs versus NS-SWNs and their distribution across different regions associated with the genes. (**e**) Plot showing methylation status (average beta value) of LINE-1 and ALU repetitive elements in SWNTS-SWNs and NS-SWN cases. (**f**) Plot showing the fold change in expression of *TET1, TET2, DNMT1* and D*NMT3A* in SWNTS-SWNs (N=24) versus NS-SWNs (N=41). (**g**) Volcano plot showing the methylation status of top variable CpGs in germline *LZTR1*-mutant (N=17) versus *LZTR1*-wildtype (N=25) SWNTS-SWNs. (**h**) Unsupervised hierarchical clustering of the top 2,000 most variably methylated CpGs in SWNTS-SWNs (N=88). Color codes denote tumor type, germline mutation status, somatic mutations in NF2, tumor location, painful tumors, and patient sex.

Online resource figure 2. Landscape of somatic alterations in SWNTS-SWNs. (**a**) TMB in SWNTS-SWNs relative to other tumor types based on WES analysis. **(b**) Variant allele frequency and distribution of somatic single nucleotide variants in *NF2* gene in SWNTS-SWNs and NS-SWNs, and germline mutations in *LZTR1* and *SMARCB1* in SWNTS. (**c**) Integrated genomic view of copy number alterations across

the whole genome of SWNTS-SWNs samples based on WGS data. (**d**) Compound CNV plot generated based on DNA methylation data showing more extensive alterations in SWNTS-SWNs versus NS-SWNs across the genome. (**e**) Oncoprint showing top most frequently deleted genes on 22q and the plot to the right shows log2 fold change in expression of these genes in SWNTS-SWNs (N=24) versus NS-SWNs (N=41). * denotes P<0.01. (**f**) Plots showing arm-level copy number changes in individual SWNTS-SWN samples in each of the three germline mutation groups.

Online resource figure 3. Pathways associated with key molecular alterations in SWNTS-SWNs. (**a**) GSEA enrichment plot of the RAS/MAPK pathway activation in germline *LZTR1*-mutant vs. *LZTR1*-wildtype and *NF2*-mutant vs. *NF2*-wildtype SWNTS-SWNs. (**b**) GSEA was performed to identify top deregulated cellular pathways in *LZTR1*-mutant vs. *LZTR1*-wildtype, *SMARCB1*-mutant (N=2) vs. *SMARCB1*-wildtype (N=22), and *NF2*-mutant (N=16) vs. *NF2*-wildtype (N=7). Red circles denote upregulated while blue circles denote down-regulated pathways. Overlapping pathways are shown in shared squares.

Online resource figure 4. Gene fusions in SWNs. Oncoprint showing the top in-frame and out-of-frame gene fusions identified in SWNTS-SWNs and their prevalence in NS-SWNs. Color codes denote tumor type, germline mutations status, anatomic location, patient sex, and number of somatic hits per tumor based on WES data.

Online resource figure 5. Transcriptome profile of pain related pathways in SWNTS-SWNs. (**a**) Plot showing the proportion of samples from each anatomic location associated with pain. (**b**) GSEA analysis of RAS/MAPK activation in painful (N=12) versus non-painful (N=8) SWNTS-SWNs and its downregulation in TR and SP (mid-

body, N=11) versus LE and UE (extremities, N=13). (**c**) Top enrichment scoring cellular pathways in painful versus non-painful SWNTS-SWNs and tumors from extremities versus mid-body. (**d**) Plot showing expression of pain-related genes that are significantly ($q<0.05$) up- or downregulated in painful versus non-painful tumors.

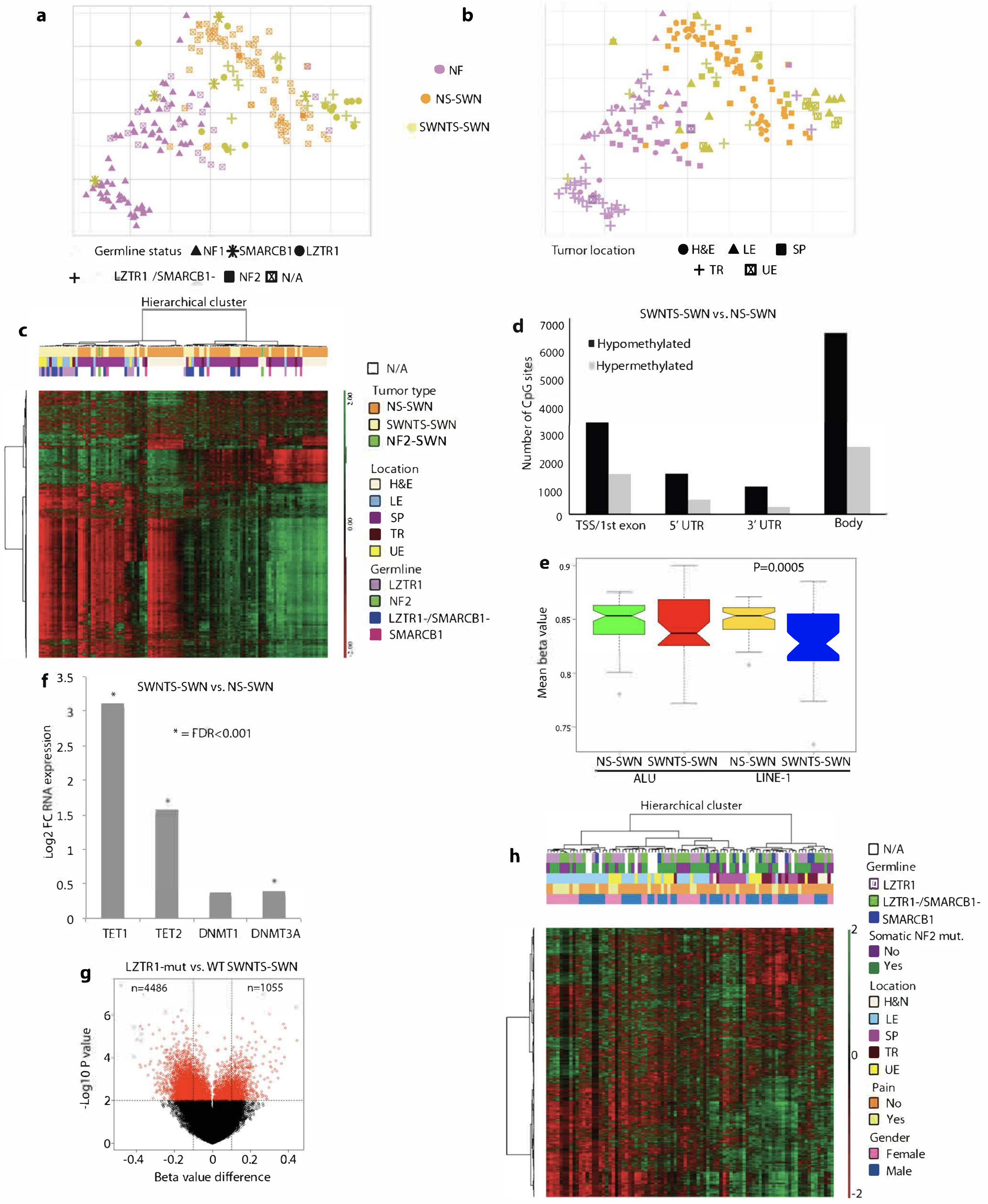
a
NF
NS-SWN
SWNTS-SWN
Germline status NF1 SMARCB1 LZTR1
LZTR1 /SMARCB1- NF2 N/A
b
Tumor location H&E LE SP
TR UE
c
Hierarchical cluster
N/A
Tumor type
NS-SWN
SWNTS-SWN
NF2-SWN
Location
H&E
LE
SP
TR
UE
Germline
LZTR1
NF2
LZTR1-/SMARCB1-
SMARCB1
d
SWNTS-SWN vs. NS-SWN
Hypomethylated
Hypermethylated
Number of CpG sites
TSS/1st exon
5′ UTR
3′ UTR
Body
e
P=0.0005
Mean beta value
NS-SWN SWNTS-SWN NS-SWN SWNTS-SWN
ALU
LINE-1
f
SWNTS-SWN vs. NS-SWN
* = FDR<0.001
Log2 FC RNA expression
TET1
TET2
DNMT1
DNMT3A
g
LZTR1-mut vs. WT SWNTS-SWN
n=4486
n=1055
-Log10 P value
Beta value difference
h
Hierarchical cluster
N/A
Germline
LZTR1
LZTR1-/SMARCB1-
SMARCB1
Somatic NF2 mut.
No
Yes
Location
H&N
LE
SP
TR
UE
Pain
No
Yes
Gender
Female
Male

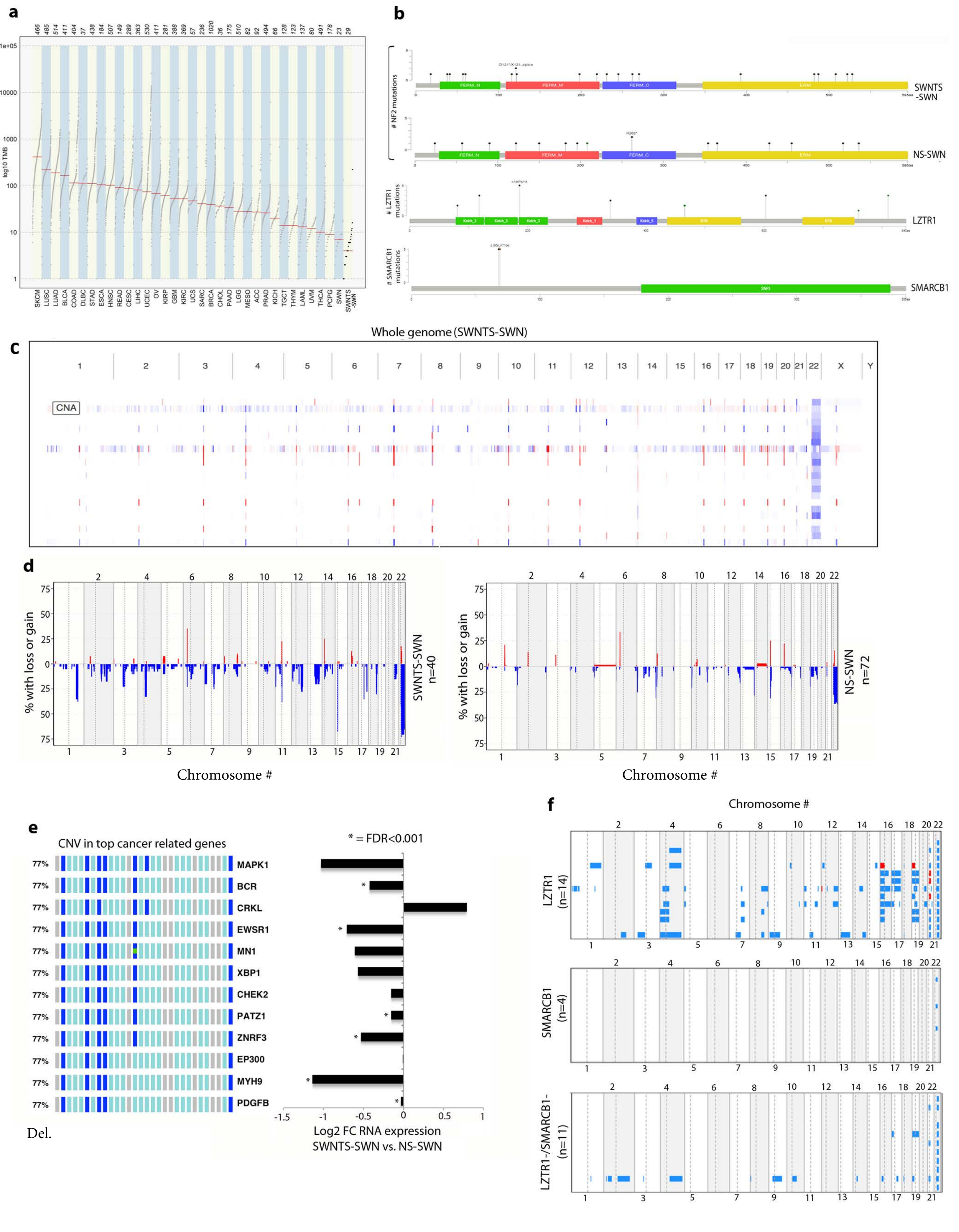
a
log10 TMB
SKCM
LUSC
LUAD
BLCA
COAD
DLBC
STAD
ESCA
HNSC
READ
CESC
LIHC
UCEC
OV
KIRP
GBM
KIRC
UCS
SARC
BRCA
CHOL
PAAD
LGG
MESO
ACC
PRAD
KICH
TGCT
THYM
LAML
UVM
THCA
PCPG
SWN
SWNTS-SWN
b
NF2 mutations
SWNTS-SWN
NS-SWN
LZTR1 mutations
LZTR1
SMARCB1 mutations
SMARCB1
Whole genome (SWNTS-SWN)
c
CNA
d
% with loss or gain
SWNTS-SWN n=40
NS-SWN n=72
Chromosome #
e
CNV in top cancer related genes
* = FDR<0.001
MAPK1
BCR
CRKL
EWSR1
MN1
XBP1
CHEK2
PATZ1
ZNRF3
EP300
MYH9
PDGFB
Del.
Log2 FC RNA expression
SWNTS-SWN vs. NS-SWN
f
Chromosome #
LZTR1 (n=14)
SMARCB1 (n=4)
LZTR1-/SMARCB1- (n=11)

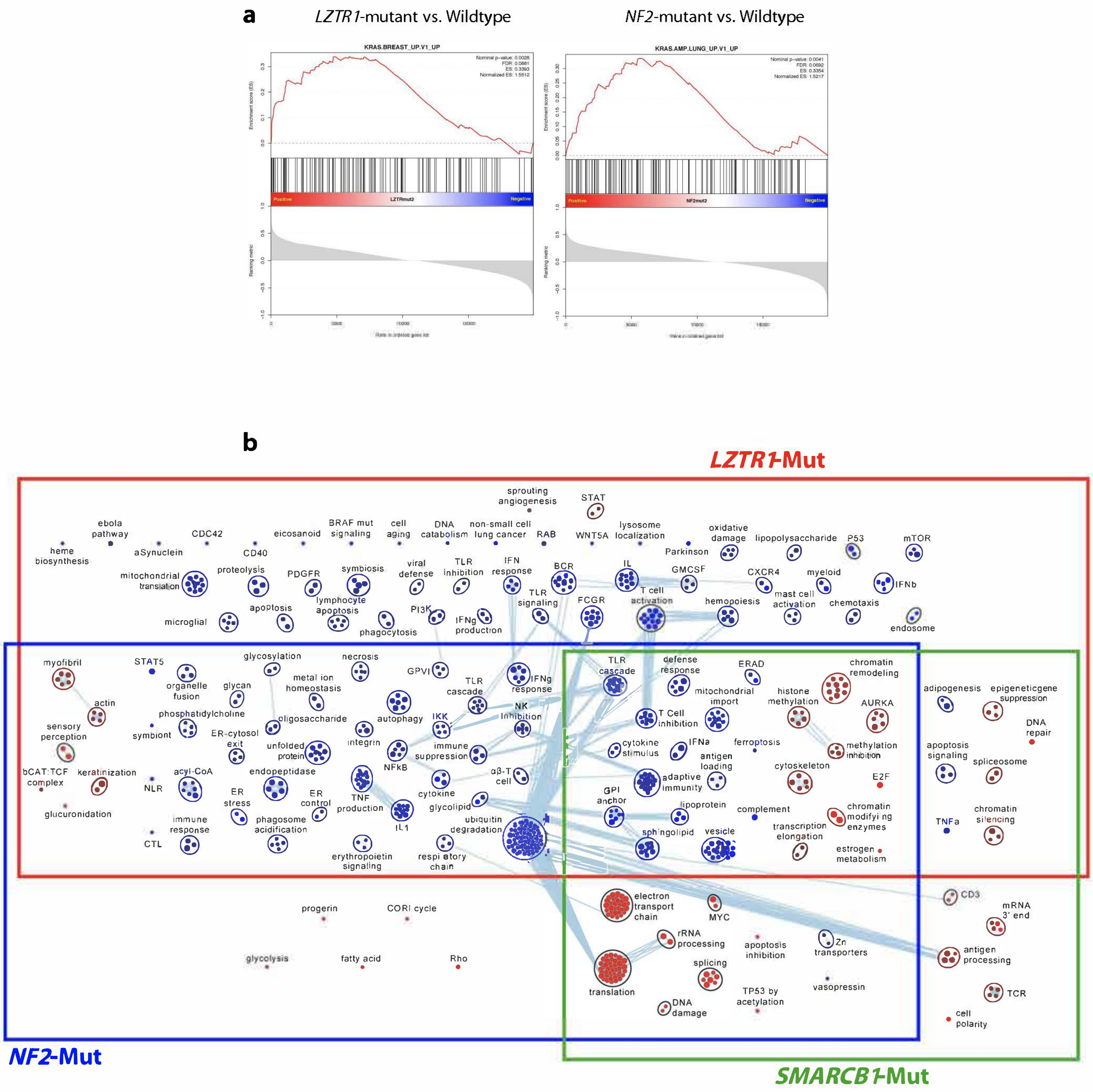
a
LZTR1-mutant vs. Wildtype
NF2-mutant vs. Wildtype
KRAS.BREAST_UP.V1_UP
KRAS.AMP.LUNG_UP.V1_UP
Positive
Negative
LZTRmut2
NF2mut2
b
LZTR1-Mut
NF2-Mut
SMARCB1-Mut
sprouting angiogenesis
STAT
ebola pathway
BRAF mut signaling
cell aging
DNA catabolism
non-small cell lung cancer
RAB
WNT5A
lysosome localization
oxidative damage
lipopolysaccharide
P53
mTOR
heme biosynthesis
aSynuclein
CDC42
eicosanoid
CD40
Parkinson
mitochondrial translation
proteolysis
PDGFR
symbiosis
viral defense
TLR inhibition
IFN response
BCR
IL
GMCSF
CXCR4
myeloid
IFNb
apoptosis
lymphocyte apoptosis
PI3K
TLR signaling
FCGR
T cell activation
hemopoiesis
mast cell activation
chemotaxis
microglial
phagocytosis
IFNg production
endosome
myofibril
STAT5
glycosylation
necrosis
GPVI
TLR cascade
IFNg response
defense response
ERAD
chromatin remodeling
organelle fusion
glycan
metal ion homeostasis
TLR cascade
NK inhibition
mitochondrial import
histone methylation
adipogenesis
epigeneticgene suppression
actin
phosphatidylcholine
oligosaccharide
autophagy
IKK
T Cell inhibition
AURKA
DNA repair
sensory perception
symbiont
ER-cytosol exit
integrin
immune suppression
cytokine stimulus
IFNa
ferroptosis
methylation inhibition
apoptosis signaling
unfolded protein
NFkB
antigen loading
spliceosome
bCAT:TCF complex
keratinization
acyl-CoA
endopeptidase
αβ-T cell
adaptive immunity
cytoskeleton
E2F
NLR
ER stress
ER control
TNF production
cytokine
GPI anchor
lipoprotein
complement
glucuronidation
glycolipid
chromatin modifying enzymes
chromatin silencing
immune response
phagosome acidification
IL1
ubiquitin degradation
transcription elongation
TNFa
CTL
sphingolipid
vesicle
erythropoietin signaling
respiratory chain
estrogen metabolism
electron transport chain
MYC
CD3
progerin
CORI cycle
mRNA 3' end
rRNA processing
apoptosis inhibition
Zn transporters
antigen processing
glycolysis
fatty acid
Rho
splicing
translation
TP53 by acetylation
vasopressin
TCR
DNA damage
cell polarity

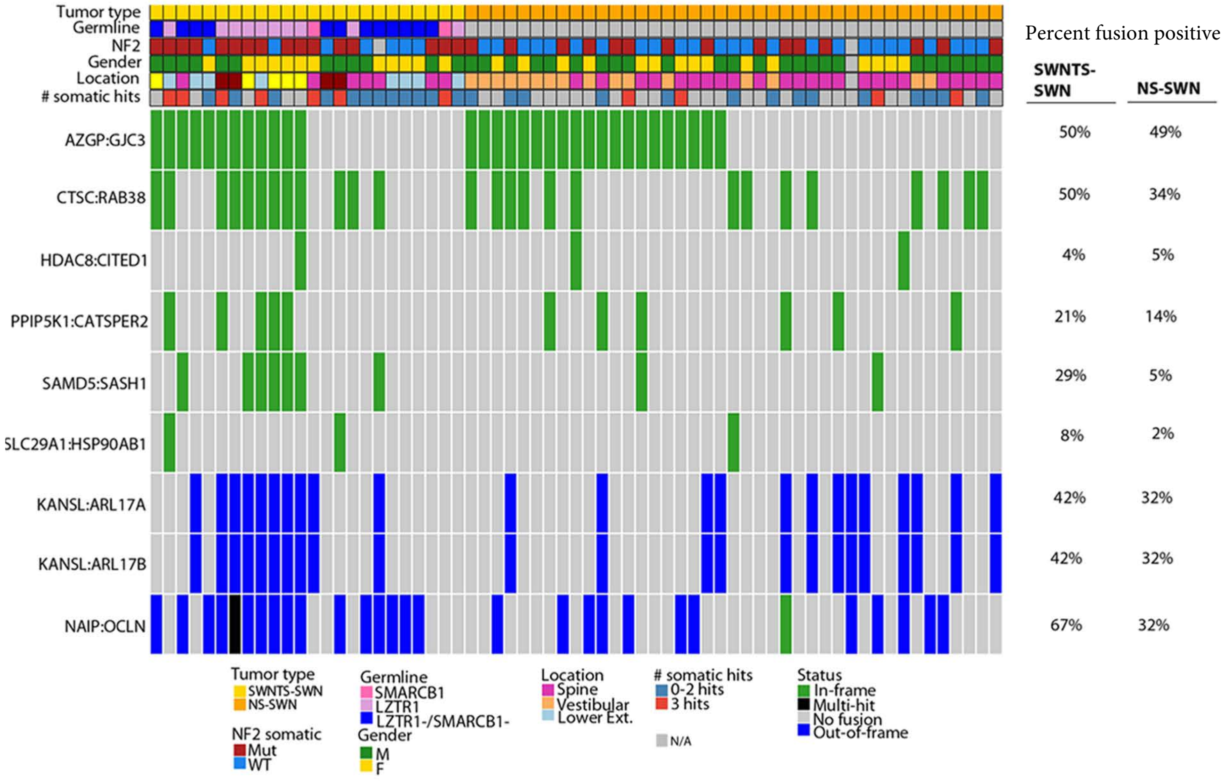

Tumor type
Germline
NF2
Gender
Location
somatic hits
Percent fusion positive
SWNTS-SWN
NS-SWN
AZGP:GJC3
50%
49%
CTSC:RAB38
50%
34%
HDAC8:CITED1
4%
5%
PPIP5K1:CATSPER2
21%
14%
SAMD5:SASH1
29%
5%
SLC29A1:HSP90AB1
8%
2%
KANSL:ARL17A
42%
32%
KANSL:ARL17B
42%
32%
NAIP:OCLN
67%
32%
Tumor type
SWNTS-SWN
NS-SWN
NF2 somatic
Mut
WT
Germline
SMARCB1
LZTR1
LZTR1-/SMARCB1-
Gender
M
F
Location
Spine
Vestibular
Lower Ext.
somatic hits
0-2 hits
3 hits
N/A
Status
In-frame
Multi-hit
No fusion
Out-of-frame

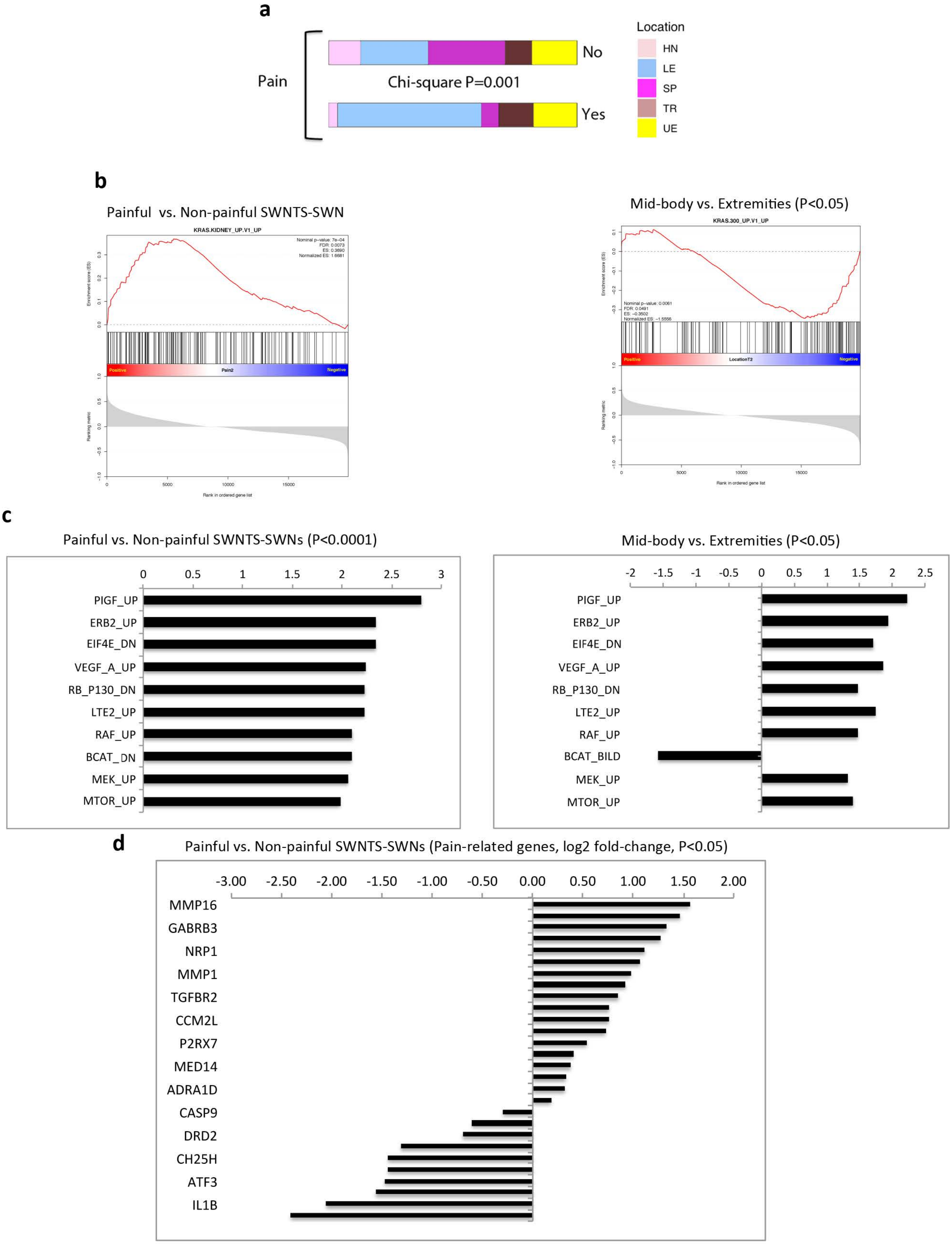

a
Pain
No
Yes
Chi-square P=0.001
Location
HN
LE
SP
TR
UE
b
Painful vs. Non-painful SWNTS-SWN
KRAS.KIDNEY_UP.V1_UP
Positive
Pain2
Negative
Rank in ordered gene list
Mid-body vs. Extremities (P<0.05)
KRAS.300_UP.V1_UP
Positive
LocationT2
Negative
Rank in ordered gene list
c
Painful vs. Non-painful SWNTS-SWNs (P<0.0001)
PIGF_UP
ERB2_UP
EIF4E_DN
VEGF_A_UP
RB_P130_DN
LTE2_UP
RAF_UP
BCAT_DN
MEK_UP
MTOR_UP
Mid-body vs. Extremities (P<0.05)
PIGF_UP
ERB2_UP
EIF4E_DN
VEGF_A_UP
RB_P130_DN
LTE2_UP
RAF_UP
BCAT_BILD
MEK_UP
MTOR_UP
d
Painful vs. Non-painful SWNTS-SWNs (Pain-related genes, log2 fold-change, P<0.05)
MMP16
GABRB3
NRP1
MMP1
TGFBR2
CCM2L
P2RX7
MED14
ADRA1D
CASP9
DRD2
CH25H
ATF3
IL1B

Online Resource Tables

Online resource table 1. Description of the cohort and molecular profiling platforms

Patient ID	Tumor ID	Center	LZTR1+, SMARCB1 +	Location	Gender	Family history	Tissue (frozen/ FFPE)	NF2 (Somatic, Sanger)	22q LOH	Fusion (SH3PXD2A-HTRA1)	Pain	Meth	WGS	WES	RNAseq	RT-PCR
1	11	UF	SMARCB1	LE	M	Sporadic	frozen	y	Y	N	Y	Y	Y	Y	N	N
1	8	UF	SMARCB1	TR	M	Sporadic	frozen	N	N	N	Y	Y	N	Y	N	N
1	10	UF	SMARCB1	TR	M	Sporadic	frozen	N	N	N	Y	Y	N	Y	N	N
1	9	UF	SMARCB1	TR	M	Sporadic	frozen	y	Y	N	Y	N	Y	Y	N	N
2	12	UF	LZTR1	TR	F	Sporadic	frozen	N	Y	N	Y	N	N	Y	N	N
2	13	UF	LZTR1	TR	F	Sporadic	frozen	y	Y	N	Y	Y	N	Y	N	N
3	32	UF	LZTR1	LE	M	Familial	frozen	y	N	N	N/A	Y	Y	Y	Y	N
3	143	UF	LZTR1	SP	M	Familial	FFPE	y	N/A	N	N/A	N	N	N	N	N
4	28	UF	LZTR1	LE	M	Sporadic	FFPE	N	Y	N	Y	Y	N	Y	N	Y
4	144	UF	LZTR1	LE	M	Sporadic	FFPE	N/A	N/A	Y	N/A	N	N	N	N	N
4	27	UF	LZTR1	SP	M	Sporadic	FFPE	y	Y	Y	Y	N	N	N	N	Y
5	114	UF	neg	HN	M	Sporadic	FFPE	N/A	N/A	N/A	N	N	N	N	N	Y
5	113	UF	neg	UE	M	Sporadic	FFPE	N/A	N/A	N/A	N	N	N	N	N	Y
6	40	UF	neg	LE	F	Sporadic	FFPE	N	Y	N	N/A	Y	N	N	N	N
6	41	UF	neg	LE	F	Sporadic	FFPE	N	Y	N	N/A	Y	N	N	N	N
6	15	UF	neg	LE	F	Sporadic	frozen	N	Y	N	N/A	Y	N	N	N	N
6	42	UF	neg	LE	F	Sporadic	FFPE	N	N/A	N	N/A	Y	N	N	N	N
6	14	UF	neg	LE	F	Sporadic	frozen	N	Y	N	N/A	Y	N	Y	N	N
7	16	UF	neg	UE	M	Sporadic	frozen	y	Y	N	N	Y	Y	Y	N	N
8	21	UF	neg	SP	F	Sporadic	frozen	N	Y	N	N/A	Y	N	N	N	N
8	30	UF	neg	UE	F	Sporadic	frozen	y	Y	N	Y	Y	Y	Y	N	N
9	20	UF	LZTR1	LE	F	Sporadic	FFPE	N	Y	N	Y	N	N	N	N	Y
9	19	UF	LZTR1	LE	F	Sporadic	FFPE	N	Y	N	Y	Y	N	N	N	Y
9	18	UF	LZTR1	LE	F	Sporadic	FFPE	y	Y	N	Y	Y	N	N	N	Y
9	17	UF	LZTR1	LE	F	Sporadic	FFPE	y	Y	N/A	Y	N	N	N	N	Y
9	145	UF	LZTR1	LE	F	Sporadic	FFPE	y	Y	Y	N/A	N	N	N	N	N
10	109	UF	LZTR1	LE	F	Sporadic	frozen	y	Y	N	Y	Y	N	Y	Y	N
10	111	UF	LZTR1	UE	F	Sporadic	frozen	y	N/A	N	Y	Y	N	Y	Y	N
10	112	UF	LZTR1	UE	F	Sporadic	frozen	y	N/A	N	Y	Y	N	Y	Y	N
10	110	UF	LZTR1	UE	F	Sporadic	frozen	N	Y	N	Y	Y	Y	Y	Y	N

10	108	UF	LZTR1	UE	F	Sporadic	frozen	y	N/A	N	Y	Y	Y	Y	Y	N
11	25	UF	SMARCB1	LE	F	Sporadic	FFPE	N	Y	N	N	Y	N	N	N	Y
11	26	UF	SMARCB1	TR	F	Sporadic	FFPE	y	Y	N	N/A	Y	N	N	N	Y
12	23	UF	LZTR1	TR	F	Sporadic	FFPE	y	Y	Y	N/A	Y	N	N	N	N
12	22	UF	LZTR1	UE	F	Sporadic	FFPE	N	Y	N		N	N	N	N	N
13	96	UF	LZTR1	SP	F	Familial	FFPE	y	Y	N	Y	N	N	N	N	Y
14	97	UF	LZTR1	LE	M	Familial	FFPE	y	Y	Y	Y	N	N	N	N	Y
14	146	UF	LZTR1	SP	M	Familial	FFPE	y	Y	N	N/A	N	N	N	N	N
15	37	UF	LZTR1	HN	M	Familial	FFPE	y	Y	N	N	N	N	N	N	Y
15	36	UF	LZTR1	SP	M	Familial	FFPE	y	Y	N	N	N	N	N	N	Y
15	38	UF	LZTR1	TR	M	Familial	FFPE	y	Y	Y	N	N	N	N	N	Y
16	24	UF	neg	TR	F	Sporadic	FFPE	N	N	N	N/A	N	N	N	N	N
17	39	UF	SMARCB1	LE	F	Familial	FFPE	y	Y	N	Y	N	N	N	N	Y
17	31	UF	SMARCB1	SP	F	Familial	frozen	y	Y	N	N	Y	Y	Y	Y	N
18	45	UF	neg	HN	F	Sporadic	FFPE	N	Y	N	N/A	N	N	N	N	N
18	44	UF	neg	LE	F	Sporadic	FFPE	y	Y	N	Y	Y	N	N	N	Y
18	43	UF	neg	TR	F	Sporadic	FFPE	N	Y	N	N/A	Y	N	N	N	N
19	29	UF	neg	SP	M	Sporadic	frozen	y	N	N	N/A	Y	Y	Y	Y	N
20	61	UF	LZTR1	SP	M	Sporadic	FFPE	y	Y	N	N	N	N	N	N	Y
20	147	UF	LZTR1	SP	M	Sporadic	FFPE	y	Y	Y	N/A	N	N	N	N	N
21	53	UF	LZTR1	LE	M	Sporadic	FFPE	y	Y	N	Y	N	N	N	N	N
21	55	UF	LZTR1	LE	M	Sporadic	FFPE	y	Y	N	Y	Y	N	N	N	Y
21	54	UF	LZTR1	LE	M	Sporadic	FFPE	N/A	N/A	N	Y	N	N	N	N	Y
21	50	UF	LZTR1	LE	M	Familial	FFPE	N	Y	Y	Y	N	N	N	N	Y
21	49	UF	LZTR1	LE	M	Familial	FFPE	y	Y	Y	Y	N	N	N	N	Y
21	52	UF	LZTR1	LE	M	Familial	FFPE	N	N/A	Y	Y	N	N	N	N	Y
21	51	UF	LZTR1	LE	M	Familial	FFPE	N	N/A	N/A	Y	N	N	N	N	Y
21	149	UF	LZTR1	LE	M	Familial	FFPE	N	Y	N	N/A	N	N	N	N	N
21	148	UF	LZTR1	LE	M	Familial	FFPE	N	Y	Y	N/A	N	N	N	N	N
22	92	UF	SMARCB1	SP	F	Sporadic	FFPE	y	Y	N	N	Y	N	N	N	Y
22	91	UF	SMARCB1	TR	F	Sporadic	FFPE	N	N/A	N	N	Y	N	N	N	Y
22	93	UF	SMARCB1	UE	F	Sporadic	FFPE	y	N/A	N	N	Y	N	N	N	Y
23	46	UF	LZTR1	LE	M	Sporadic	FFPE	y	Y	N	N	Y	N	N	N	Y
23	48	UF	LZTR1	LE	M	Sporadic	FFPE	N	N/A	N	N	N	N	N	N	Y
23	47	UF	LZTR1	LE	M	Sporadic	FFPE	y	Y	N	N	Y	N	N	N	Y
24	73	UF	neg	HN	M	Sporadic	FFPE	N	N	N	N/A	N	N	N	N	Y

24	72	UF	neg	SP	M	Sporadic	FFPE	y	Y	Y	N/A	N	N	N	N	Y
25	56	UF	neg	SP	F	Sporadic	frozen	y	N	N	N	Y	Y	Y	N	N
26	59	UF	SMARCB1	UE	F	Familial	FFPE	y	Y	N	Y	N	N	N	N	Y
27	87	UF	LZTR1	HN	F	Sporadic	FFPE	N	Y	N	N	Y	N	N	N	Y
27	88	UF	LZTR1	UE	F	Sporadic	FFPE	y	Y	N	N	Y	N	Y	N	Y
28	63	UF	LZTR1	LE	F	Sporadic	frozen	y	N	N	Y	Y	N	N	N	N
28	62	UF	LZTR1	LE	F	Sporadic	frozen	y	Y	N	Y	Y	N	N	N	N
28	64	UF	LZTR1	LE	F	Sporadic	frozen	y	Y	N	Y	Y	N	N	N	N
28	65	UF	LZTR1	LE	F	Sporadic	frozen	y	Y	N	Y	Y	N	N	N	N
28	76	UF	LZTR1	LE	F	Sporadic	FFPE	y	Y	N	N	Y	N	N	N	Y
28	77	UF	LZTR1	LE	F	Sporadic	FFPE	y	Y	N	N	Y	N	N	N	Y
28	80	UF	LZTR1	LE	F	Sporadic	FFPE	y	Y	N	Y	N	N	N	N	Y
28	78	UF	LZTR1	LE	F	Sporadic	FFPE	y	Y	Y	Y	N	N	N	N	Y
28	79	UF	LZTR1	LE	F	Sporadic	FFPE	y	Y	Y	Y	N	N	N	N	Y
29	66	UF	neg	LE	M	Sporadic	FFPE	N/A	N/A	N	N/A	N	N	N	N	Y
30	67	UF	LZTR1	HN	M	Sporadic	FFPE	y	N/A	N	N	N	N	N	N	Y
31	68	UF	neg	HN	M	Sporadic	FFPE	N	N	N	N	Y	N	N	N	Y
31	69	UF	neg	HN	M	Sporadic	FFPE	N	Y	N	N	Y	N	N	N	Y
31	70	UF	neg	HN	M	Sporadic	FFPE	N	Y	N	N	Y	N	Y	N	Y
31	71	UF	neg	SP	M	Sporadic	FFPE	N	Y	N	N	Y	N	N	N	Y
32	75	UF	neg	LE	M	Sporadic	FFPE	y	Y	N	N	Y	N	Y	N	Y
32	74	UF	neg	TR	M	Sporadic	FFPE	N	Y	N	N	Y	N	N	N	Y
33	102	UF	neg	LE	M	Sporadic	frozen	y	Y	N	N	Y	N	Y	N	N
33	94	UF	neg	LE	M	Sporadic	FFPE	y	Y	N	N	Y	N	N	N	Y
34	81	UF	neg	LE	M	Sporadic	frozen	N	Y	N	N/A	Y	N	Y	N	N
35	83	UF	LZTR1	SP	F	Sporadic	FFPE	N/A	N/A	N	N	N	N	N	N	Y
35	82	UF	LZTR1	SP	F	Sporadic	FFPE	N	N/A	Y	N	N	N	N	N	Y
36	100	UF	LZTR1	UE	M	Familial	FFPE	y	Y	N	Y	Y	N	N	N	N
37	85	UF	neg	LE	F	Sporadic	FFPE	N/A	N/A	N	Y	N	N	N	N	Y
37	86	UF	neg	LE	F	Sporadic	FFPE	N/A	N/A	N	Y	N	N	N	N	Y
37	84	UF	neg	TR	F	Sporadic	FFPE	N/A	N/A	N	Y	N	N	N	N	Y
38	90	UF	LZTR1	TR	M	Sporadic	frozen	y	N	N	N/A	Y	Y	N	Y	N
38	89	UF	LZTR1	TR	M	Sporadic	frozen	y	Y	N	N/A	Y	N	Y	Y	N
39	6	UF	LZTR1	HN	M	Sporadic	frozen	y	Y	N	N/A	Y	N	Y	N	N
39	95	UF	LZTR1	LE	M	Sporadic	frozen	y	Y	N	N/A	Y	Y	Y	N	N
40	106	UF	SMARCB1	UE	M	Sporadic	FFPE	y	Y	N		Y	N	N	N	N

41	105	UF	neg	UE	M	Sporadic	frozen	y	Y	N	N/A	Y	N	Y	N	N
41	103	UF	neg	N/A	M	Sporadic	FFPE	N/A	N/A	N	N/A	N	N	N	N	Y
41	104	UF	neg	N/A	M	Sporadic	FFPE	N/A	N/A	N	N/A	N	N	N	N	Y
42	101	UF	LZTR1	UE	F	Familial	FFPE	N	N/A	N	N/A	N	N	N	N	Y
43	150	UF	neg	HN	M	Sporadic	FFPE	N	Y	Y	N/A	N	N	N	N	N
43	107	UF	neg	SP	M	Sporadic	FFPE	N	N	Y	N/A	N	N	N	N	N
44	98	UF	LZTR1	TR	F	Sporadic	FFPE	N/A	N/A	N/A	Y	N	N	N	N	Y
44	99	UF	LZTR1	TR	F	Sporadic	FFPE	N/A	N/A	N/A	Y	N	N	N	N	Y
45	141	UF	neg	LE	M	Sporadic	frozen	y	N	N/A	N	N	Y	N	N	N
45	139	UF	neg	UE	M	Sporadic	frozen	y	Y	N/A	Y	N	Y	N	N	N
45	140	UF	neg	UE	M	Sporadic	frozen	y	Y	N/A	Y	N	Y	N	N	N
46	142	UF	SMARCB1	LE	F	Familial	frozen	N/A	N/A	N/A	Y	N	Y	N	N	N
47	7	UF	neg	LE	F	Sporadic	frozen	y	Y	N	N/A	Y	N	Y	N	N
47	151	UF	neg	UE	F	Sporadic	FFPE	N/A	N/A	N	N/A	N	N	N	N	N
48	34	UF	SMARCB1	HN	F	Familial	FFPE	y	Y	Y	Y	N	N	N	N	Y
48	33	UF	SMARCB1	HN	F	Familial	FFPE	N/A	N/A	N/A	Y	N	N	N	N	Y
48	152	UF	SMARCB1	TR	F	Familial	FFPE	y	N/A	N	N/A	N	N	N	N	N
48	35	UF	SMARCB1	N/A	F	Familial	FFPE	N/A	Y	N	Y	N	N	N	N	Y
49	58	UF	SMARCB1	SP	F	Familial	FFPE	y	Y	N	N	N	N	N	N	Y
49	57	UF	SMARCB1	SP	F	Familial	FFPE	N/A	N/A	N	N	N	N	N	N	Y
49	153	UF	SMARCB1	TR	F	Familial	FFPE	N	Y	Y	N/A	N	N	N	N	N
50	60	UF	SMARCB1	SP	M	Familial	FFPE	N	Y	N	N	N	N	N	N	Y
50	154	UF	SMARCB1	TR	M	Familial	FFPE	N	Y	N	N/A	N	N	N	N	N
50	155	UF	SMARCB1	TR	M	Familial	FFPE	N	Y	N	N/A	N	N	N	N	N
51	1	Argentina	neg	LE	F	Sporadic	frozen	N	N	N	Y	Y	N	Y	Y	N
51	2	Argentina	neg	LE	F	Sporadic	frozen	N	N	N	Y	Y	N	Y	Y	N
51	3	Argentina	neg	LE	F	Sporadic	frozen	N	N	N	Y	Y	N	Y	Y	N
51	4	Argentina	neg	LE	F	Sporadic	frozen	N	N	N	Y	Y	N	Y	Y	N
52	117	UHN	LZTR1	LE	M	Sporadic	FFPE	N/A	N/A	N	N	Y	N	N	N	N
52	119	UHN	LZTR1	LE	M	Sporadic	frozen	y	Y	N	Y	Y	N	Y	Y	N
53	132	UHN	neg	LE	M	Sporadic	FFPE	N/A	N/A	N	Y	N	N	N	N	N
53	115	UHN	neg	UE	M	Sporadic	FFPE	N/A	N/A	N	N	Y	N	N	N	N
53	130	UHN	neg	UE	M	Sporadic	FFPE	N/A	N/A	N	Y	Y	N	N	N	N
54	127	UHN	neg	SP	M	Sporadic	FFPE	N/A	Y	N	N	Y	N	N	N	N
54	131	UHN	neg	SP	M	Sporadic	FFPE	N/A	Y	N	N	Y	N	N	N	N
54	129	UHN	neg	SP	M	Sporadic	FFPE	N/A	N/A	N	N	Y	N	N	N	N

54	135	UHN	LZTR1	SP	M	Sporadic	frozen	y	N	N	N	Y	Y	Y	Y	N
55	122	UHN	neg	SP	M	Sporadic	frozen	y	Y	N	N	Y	N	Y	Y	N
55	118	UHN	neg	UE	M	Sporadic	frozen	y	N/A	N	N	Y	N	Y	Y	N
56	126	UHN	neg	UE	F	Sporadic	FFPE	N/A	Y	N	N	Y	N	N	N	N
57	124	UHN	neg	LE	M	Sporadic	FFPE	N/A	N/A	N	N	Y	N	N	N	N
57	137	UHN	neg	UE	M	Sporadic	FFPE	N/A	Y	N	N	Y	N	N	N	N
58	138	UHN	neg	UE	M	Sporadic	FFPE	N/A	Y	N	N	Y	N	N	N	N
58	134	UHN	neg	UE	M	Sporadic	FFPE	N/A	N/A	N	N	Y	N	N	N	N
59	136	UHN	neg	SP	M	Sporadic	frozen	N	Y	N	Y	Y	Y	Y	Y	N
60	5	UHN	neg	SP	F	Sporadic	frozen	N/A	N	N	N	Y	N	N	Y	N
61	128	UHN	neg	LE	M	Sporadic	FFPE	N/A	Y	N	N	Y	N	N	N	N
61	116	UHN	neg	LE	M	Sporadic	FFPE	N/A	N/A	N	N	Y	N	N	N	N
62	120	UHN	SMARCB1	SP	F	Sporadic	frozen	y	Y	N	Y	Y	N	Y	Y	N
63	123	UHN	neg	LE	M	Sporadic	frozen	y	N/A	N	N	Y	Y	Y	Y	N
63	133	UHN	neg	TR	M	Sporadic	FFPE	N/A	N/A	N	N	Y	N	N	N	N
64	125	UHN	neg	TR	M	Sporadic	frozen	y	Y	N	N	Y	Y	Y	Y	N
64	121	UHN	neg	TR	M	Sporadic	frozen	N	N/A	N	N	Y	Y	Y	Y	N
65	156	UF	LZTR1	SP	M	Sporadic	FFPE	y	N/A	N	N/A	N	N	N	N	N
65	157	UF	LZTR1	SP	M	Sporadic	FFPE	y	N/A	Y	N/A	N	N	N	N	N
66	158	UF	neg	HN	M	Familial	FFPE	N	Y	N	N/A	N	N	N	N	N
67	159	UF	neg	LE	M	Sporadic	FFPE	y	N/A	N	N/A	N	N	N	N	N
68	160	UF	LZTR1	SP	F	Sporadic	FFPE	N/A	N/A	N	N/A	N	N	N	N	N
68	161	UF	LZTR1	SP	F	Sporadic	FFPE	N/A	N/A	N	N/A	N	N	N	N	N
69	162	UF	neg	SP	M	Sporadic	FFPE	N/A	N/A	Y	N/A	N	N	N	N	N
70	163	UF	neg	N/A	F	Familial	FFPE	N/A	N/A	Y	N/A	N	N	N	N	N
71	164	UF	neg	SP	F	Sporadic	FFPE	N	N/A	N	N/A	N	N	N	N	N
72	165	UF	LZTR1	N/A	M	Familial	FFPE	N/A	N/A	N/A	N/A	N	N	N	N	N

3 **Online resource table 2.** Top 50 differentially methylated CpGs in SWNTS-SWNs vs. NS-SWNs

ID	CHR	Gene Symbol	RELATION_TO_UCSC_CPG_ISLAND	UCSC_REFGENE_GROUP.0	Pvalue	pp.adj	Mean diff (SWN-SWNTS)
cg09698465	12		Island		1.41E-32	1.18E-30	0.609206
cg22876699	3				8.14E-98	3.64E-94	0.609195
cg02154531	X	NHSL2	N_Shore	Body	3.20E-75	3.27E-72	0.608366
cg26175287	14	C14orf139		TSS1500	1.19E-89	3.01E-86	0.597191
cg10506618	3	TRAK1	S_Shelf	Body	1.61E-87	3.39E-84	0.584945
cg05128056	15		N_Shore		9.93E-72	8.32E-69	0.561456
cg13851870	19	CCDC124	Island	5'UTR	1.98E-87	4.12E-84	0.561247

cg17189020	17	ATP2A3	S_Shore	Body	2.69E-57	1.04E-54	0.550251
cg25152348	22	NCAPH2	Island	1stExon	7.32E-90	1.88E-86	0.533018
cg12026095	19	FTL	Island	TSS200	3.83E-108	4.26E-104	0.525319
cg13003239	3	WDR6	Island	TSS200	2.76E-108	3.32E-104	0.522194
cg08206623	11	CDKN1C	Island	TSS1500	8.85E-76	9.29E-73	0.514188
cg03479491	22	TTLL8	N_Shore	TSS1500	3.40E-90	8.93E-87	0.510783
cg00769882	1	HSPA6	N_Shore	TSS1500	1.80E-49	4.60E-47	0.504404
cg27196695	10	INPP5A		Body	3.00E-48	7.15E-46	0.497984
cg03543448	16	GLIS2		Body	4.21E-54	1.37E-51	0.496372
cg06669598	6	ECHDC1		3'UTR	2.21E-60	1.00E-57	0.496009
cg13525026	17	MYO15A		Body	1.33E-60	6.12E-58	0.494059
cg16646879	16		N_Shore		9.66E-79	1.16E-75	0.483687
cg20144008	2		Island		1.36E-83	2.31E-80	0.480808
cg12416290	3				4.31E-106	3.91E-102	0.480693
cg07060261	13				8.71E-89	2.01E-85	0.480298
cg01938825	7				1.50E-29	9.98E-28	0.478751
cg01684248	16		N_Shelf		1.12E-75	1.17E-72	0.468001
cg07576219	1	ACOT11	S_Shelf	TSS1500	5.34E-69	3.85E-66	0.466562
cg20149170	1	EPHB2		Body	2.04E-40	2.99E-38	0.464245
cg18048953	12		N_Shelf		1.71E-74	1.69E-71	0.453483
cg26651188	12	LPCAT3		Body	3.87E-46	8.20E-44	0.448319
cg08772111	2	HDAC4	N_Shelf	Body	5.42E-52	1.58E-49	0.446714
cg03159409	12	FAM101A	N_Shore	5'UTR	3.64E-28	2.15E-26	0.445728
cg01904886	12				2.46E-43	4.34E-41	0.435614
cg07515565	6	GMDS	Island	3'UTR	8.02E-32	6.35E-30	0.430926
cg14028622	4		N_Shore		3.26E-61	1.55E-58	0.42941
cg11902748	20		S_Shore		6.22E-60	2.74E-57	0.428732
cg07170824	12	ACVRL1	Island	1stExon	7.80E-45	1.51E-42	0.428394
cg03768916	10	ARHGAP22	Island	TSS200	1.11E-87	2.41E-84	0.427959
cg11075029	15	SHF	S_Shore	5'UTR	1.89E-34	1.82E-32	0.427369
cg05967295	7	SMURF1	Island	1stExon	3.63E-54	1.19E-51	0.426797
cg26523866	1				2.74E-44	5.10E-42	0.426322
cg06947608	10	C10orf90		3'UTR	8.27E-45	1.60E-42	0.423621
cg05986007	5				5.80E-62	2.86E-59	0.422774
cg07481360	3	DLG1		Body	1.24E-42	2.09E-40	0.420483
cg05204798	11	MPPED2	Island	1stExon	1.17E-88	2.66E-85	0.412241
cg22538757	12				1.55E-29	1.03E-27	0.411849
cg01713095	4	ABCE1		3'UTR	9.88E-49	2.43E-46	0.411815
cg11495544	17	GRB2	S_Shore	TSS1500	2.59E-36	2.89E-34	0.411712
cg20980653	6	COL11A2		Body	1.51E-34	1.47E-32	0.411626

cg25840318	4				1.62E-22	5.97E-21	0.408842
cg02913521	2	ARHGEF4	Island	Body	3.71E-73	3.40E-70	0.407339

4 **Online resource table 3.** Cosmic signatures in SWNTS-SWNs and NS-SWNs

**SWNTS-SWN**						
Given tumor ID	Signature.1A	Signature.1B	Signature.2	Signature.6	Signature.15	Signature.20
67	0	0	0	0	1	0
13	0	0	0.232237435	0	0	0
129	0	0	0	0	0	0
31	0.244648679	0	0.144203055	0.517653227	0	0
30	0.2677777	0	0	0.614178261	0	0
112	0.353869738	0	0.067998603	0.102731828	0	0
89	0.527848752	0	0	0.086183474	0.385967775	0
105	0.587312105	0	0	0	0.412687895	0
95	0.613756231	0	0	0	0.386243769	0
108	0.62390823	0	0	0.308910637	0	0.067181133
32	0.634581552	0	0	0.365418448	0	0
9	0.695786688	0	0	0.093327566	0	0
136	0.737792003	0	0	0	0.225017884	0
110	0.971872209	0	0	0	0	0
28	1	0	0	0	0	0
8	1	0	0	0	0	0
11	1	0	0	0	0	0
12	1	0	0	0	0	0
109	1	0	0	0	0	0
111	1	0	0	0	0	0
6	1	0	0	0	0	0
7	1	0	0	0	0	0
121	1	0	0	0	0	0
125	1	0	0	0	0	0
123	1	0	0	0	0	0
16	1	0	0	0	0	0
29	1	0	0	0	0	0
56	1	0	0	0	0	0
81	1	0	0	0	0	0
**NS-SWN**						
Given tumor ID	Signature.1A	Signature.1B	Signature.2	Signature.6	Signature.15	Signature.20
1	1	0	0	0	0	0
2	0	0	0	0	0.726384057	0
3	0	0	0	0	0.931999368	0

4	0	0	0	0	0.523427928	0
5	0.119553995	0	0	0	0	0.216622177
6	1	0	0	0	0	0
7	0.634581552	0	0	0.365418448	0	0
8	0.454983231	0	0	0.151514914	0	0.234442061
9	0.972873985	0	0	0	0	0
10	0.760620752	0	0	0.207898926	0	0
11	0.155134481	0	0	0	0	0.280862457
12	1	0	0	0	0	0
13	0.128204941	0	0	0	0.58183193	0
14	0.957102586	0	0	0	0	0
15	1	0	0	0	0	0
16	0.974515659	0	0	0	0	0
17	0.282107737	0.182593459	0	0.112263669	0	0
18	0	0	0	0	1	0
19	0.620837743	0	0	0	0.379162231	0
20	0.185788861	0	0	0.272521379	0.190938895	0
21	0.749829211	0	0	0	0	0
22	0.791281342	0	0	0	0	0
23	0.577027219	0	0	0.39906762	0	0

Online resource table 4. Sanger-validated variants and filtered variants from WES and WGS

Patient ID	Germline mutation	Tumor	CHR	Genomic position	Gene	Consequence	DNA alteration	Effect
28	*LZTR1*	65	3	113765568	*KIAA1407*	frameshift	c.141dupA	p.Ala48Serfs*13
			X	123654474	*ODZ1*	missense	c.3194G>A	p.Arg1065Gln
			22	30069483	*NF2*	splicing	c.241-13T>A	r.spl?
			4	184203857	*WWC2*	splicing	c.2685-4A>G	r.spl?
		64	1	6204076	*CHD5*	splicing	c.1934+8G>A	r.spl?
			1	54666181	*MRPL37*	missense	c.265C>A	p.Arg89Ser
			3	113765568	*KIAA1407*	frameshift	c.141dupA	p.Ala48Serfs*13
			6	47847013	*PTCHD4*	missense	c.1567G>A	p.Val523Ile
			7	124511062	*POT1*	missense	c.158C>T	p.Thr53Ile
			16	67910446	*EDC4*	nonsense	c.295G>T	p.Glu99*
			22	30074228	*NF2*	frameshift	c.1241delG	p.Ser414Thrfs*18
		63	3	113765568	*KIAA1407*	frameshift	c.141dupA	p.Ala48Serfs*13
			22	30074313	*NF2*	splicing	c.1325+1G>T	r.spl?
			X	123654474	*ODZ1*	missense	c.3194G>A	p.Arg1065Gln
		62	3	113765568	*KIAA1407*	frameshift	c.141dupA	p.Ala48Serfs*13

			22	30064372	*NF2*	frameshift	c.687_688delAG	p.Lys229Asnfs*19
			9	132482974	*PRRX2*	missense	c.547G>A	p.Ala183Thr
3	*LZTR1*	32	22	30069483	*NF2*	splicing	c.1340+8G>T	r.spl?
			X	103268025	*H2BFWT*	missense	c.208C>T	p.Arg70Cys
23	*LZTR1*	46	1	99772184	LPPR4	missense	c.1910C>T	p.Pro637Leu
			22	30032794	NF2	nonsense	c.169C>T	p.Arg57*
		47	22	30069324	NF2	frameshift	c.1189delC	p.Leu397Phefs*29
27	*LZTR1*	88	1	200818596	CAMSAP2	missense	c.2732G>A	p.Arg911His
			22	30000102	NF2	splicing	c.114+1G>A	r.spl?
			22	30067899	NF2	nonsense	c.1084C>T	p.Gln362*
38	*LZTR1*	89	3	43618294	ANO10	missense	c.1052G>A	p.Gly351Glu
			20	30915460	KIF3B	missense	c.1964A>G	p.Tyr655Cys
			15	42727650	ZNF106	missense	c.4744A>T	p.Thr1582Ser
			2	3660954	COLEC11	missense	c.226G>A	p.Gly76Ser
			1	109192852	HENMT1	missense	c.737A>T	p.Asp246Val
			22	30057253	NF2	frameshift	c.735delC	p.Pro246Leufs*5
			1	225519211	DNAH14	missense	c.9517C>T	p.Arg3173Trp
			15	81201507	CEMIP	missense	c.1657G>A	p.Gly553Ser
10	*LZTR1*	108	22	30032747	NF2	nonsense	c.122G>A	p.Trp41*
			7	94248102	SGCE	missense	c.738G>C	p.Arg246Ser
		109	22	30077437	NF2	nonsense	c.1584C>A	p.Tyr528*
			8	145675905	CYHR1	missense	c.1072G>T	p.Val358Phe
			17	40817537	TUBG2	missense	c.650G>A	p.Arg217His
			1	220338096	RAB3GAP2	missense	c.3133T>C	p.Phe1045Leu
		110	22	30051610	NF2	nonsense	c.544G>T	p.Glu182*
			X	31089893	FTHL17	missense	c.178T>A	p.Ser60Thr
			1	153507257	S100A6	missense	c.188A>T	p.Asn63Ile
		111	22	30070931	NF2	splicing	c.1446+1G>C	r.spl?
		112	22	30057330	NF2	splicing	c.810+2T>C	r.spl?
2	*LZTR1*	12	22	30069476	NF2	splicing	c.1340+1G>A	r.spl?
			19	46891837	PPP5C	missense	c.1204G>A	p.Val402Met
		13	22	30064328	NF2	nonsense	c.892C>T	p.Gln298*
			18	32823128	ZNF397	missense	c.427C>T	p.Pro143Ser
			21	38853117	DYRK1A	missense	c.505T>C	p.Ser169Pro
			14	99866483	SETD3	missense	c.1291G>A	p.Glu431Lys
4	*LZTR1*	28	19	57955010	ZNF749	nonsense	c.494C>G	p.Ser165*
17	*SMARCB1*	31	22	30051658	*NF2*	nonsense	c.343C>T	p.Arg115*
			X	128615117	*SMARCA1*	frameshift	c.2227delG	p.Ala743Glnfs*57
			6	139097303	*CCDC28A*	missense	c.316G>A	p.Ala106Thr

			7	135106925	*CNOT4*	nonsense	c.352C>T	p.Gln118*
			19	55502038	*NLRP2*	missense	c.2697G>T	p.Leu899Phe
			19	39592142	*PAPL*	missense	c.1078C>T	p.Pro360Ser
			2	220494346	*SLC4A3*	intronic	c.529+203G>T	r.spl?
			19	39592142	ACP7	missense	c.1078C>T	p.Pro360Ser
			15	85405971	ALPK3	frameshift	c.4841_4902del	p.Gly1615fs
			2	234719540	MROH2A	missense	c.2558C>T	p.Thr850Met
			2	234719540	*HEATR7B1*	missense	c.2549C>T	p.Thr850Met
			10	69948821	*MYPN*	missense	c.2038C>T	p.Arg680Trp
11	*SMARCB1*	26	22	30038245	NF2	frameshift	c.418delC	p.L140Sfs*34
19	*LZTR1-/SMARCB1-*	29	19	52794246	*ZNF766*	missense	c.1247A>G	p.His416Arg
			22	30000041	*NF2*	frameshift	c.54delA	p.Gln18Hisfs*7
			22	30032842	*NF2*	in frame deletion	c.217_240del24	p.Ala73_Lys80del
			21	41450853	DSCAM	missense	c.4472G>A	p.R1491H
			8	23540375	*NKX3-1*	missense	c.28G>C	p.Gly10Arg
6	*LZTR1-/SMARCB1-*	14	11	57582901	*CTNND1*	missense	c.2719C>T	p.His907Tyr
			1	41483726	*SLFNL1*	missense	c.538C>T	p.Pro180Ser
			9	138649038	*KCNT1*	missense	c.580G>A	p.Val194Ile
			22	24143334	*SMARCB1*	intronic	c.500+66T>G	r.spl?
			1	156256175	*TMEM79*	nonsense	c.882C>A	p.Tyr294*
		15	22	24175822	*SMARCB1*	frameshift	c.1050_1053del	p.P351Cfs*5
8	*LZTR1-/SMARCB1-*	30	22	30057209	*NF2*	nonsense	c.442G>T	p.Glu148*
			11	124670247	MSANTD2	silent	c.30C>T	p.Pro10=
			9	32635479	TAF1L	silent	c.99C>T	p.Gly33=
			X	12939330	TLR8	missense	c.2171T>A	p.Ile724Asn
			1	64644099	*ROR1*	missense	c.2375C>T	p.Pro792Leu
			14	25443977	*STXBP6*	missense	c.48T>A	p.Asp16Glu
		21	10	117061554	*ATRNL1*	splicing	c.2818+1G>A	r.spl?
			19	4475549	*HDGFRP2*	missense	c.257A>G	p.Asn86Ser
7	*LZTR1-/SMARCB1-*	16	1	147380239	*GJA8*	missense	c.157G>A	p.Val53Met
			17	11881897	ZNF18	frameshift	c.1027delC	p.Leu343fs
			9	117168967	WHRN	missense	c.1904C>T	p.Pro635Leu
			15	88679142	NTRK3	missense	c.895C>T	p.Leu299Phe
			7	84647608	SEMA3D	missense	c.1505A>T	p.His502Leu
			9	117168967	*DFNB31*	missense	c.755C>T	p.Pro252Leu
			22	30035199	*NF2*	nonsense	c.361C>T	p.Gln121*
		6	11	10503727	AMPD3	missense	c.571G>C	p.Gly191Arg
			15	90349492	ANPEP	missense	c.323C>T	p.Thr108Ile

39	*LZTR1-/SMARCB1-*		11	105804674	GRIA4	missense	c.2273C>T	p.Thr758Met
			1	67787573	IL12RB2	splicing	c.364+1G>T	r.spl?
			22	30074302	NF2	splicing	c.1565_1574+29del	r.spl?
			8	103220387	RRM2B	missense	c.1246G>A	p.Val416Ile
			11	62751465	SLC22A6	missense	c.424A>T	p.Met142Leu
			13	38211588	TRPC4	frameshift	c.2401delC	p.Leu801fs*4
		95	17	73567795	LLGL2	missense	c.2224A>G	p.Ile742Val
			22	30069314	NF2	frameshift	c.1180_1204del24	p.(Glu394Profs*24)
			12	48458895	SENP1	frameshift	c.1227dupA	p.Gly410fs
			20	51871123	TSHZ2	nonsense	c.1126A>T	p.Lys376*
25	*LZTR1-/SMARCB1-*	56	22	30035202	NF2	splicing	c.363+1G>T	r.spl?
			22	30032794	NF2	nonsense	c.169C>T	p.Arg57*
47	*LZTR1-/SMARCB1-*	7	1	114968212	TRIM33	frameshift	1553_1554del	p.H518Qfs*38
			15	89401776	ACAN	missense	c.5960T>C	p.Leu1987Pro
			17	65014335	CACNG4	missense	c.251A>G	p.Asn84Ser
			1	111741325	DENND2D	missense	c.283C>T	p.Arg95Trp
			15	72460862	GRAMD2A	missense	c.238GT	p.Asp80Tyr
			22	30038185	NF2	splicing	c.364-6_372del15	p.Val122_Gln125del
			5	140719445	PCDHGA2	missense	c.907A>G	p.Ile303Val
			3	69117092	UBA3	missense	c.415T>C	p.Cys139Arg
33	*LZTR1-/SMARCB1-*	94	12	13722812	GRIN2B	missense	c.2311G>C	p.Gly771Arg
			22	30057302	NF2	nonsense	c.784C>T	p.Arg262*
			22	50728552	PLXNB2	frameshift	c.461_462delTG	p.Val154Glyfs*11
21		53	16	57113508	NLRC5	missense	c.5288G>A	p.Ser1763ASN
			22	30074302	NF2	nonsense	c.1564G>T	p.Glu522*

6 **Online resource table 5.** Structural variants identified using WGS dataset

	Coding/Non-coding SVs		Types of SVs						Inter/Intra chromosomal SVs		
Given tumor code	Coding	Noncoding	Deletion	Duplication	Insertion	Inversion	Inverted translocation	Translocation	Interchromosomal	Intrachromosomal	Total
6	7	76	54	12	11	2	3	1	4	79	83
95	1	66	41	12	7	5	2	0	2	65	67
9	7	62	33	13	6	3	3	11	14	55	69
11	5	40	14	16	6	2	5	2	7	38	45
16	4	12	9	2	0	0	5	0	5	11	16
29	1	52	42	6	3	0	2	0	2	51	53
30	8	53	13	17	5	7	13	6	19	42	61
31	6	36	30	5	1	2	4	0	4	38	42
32	1	48	28	11	9	0	1	0	1	48	49
56	9	71	26	42	6	1	2	3	5	75	80

90	215	628	35	71	24	671	21	21	42	801	843
108	10	47	37	7	3	4	3	3	6	51	57
110	2	76	47	10	8	5	4	4	8	70	78
139	4	58	50	4	7	0	1	0	1	61	62
140	3	68	62	3	4	0	1	1	2	69	71
141	1	35	29	2	4	1	0	0	0	36	36
142	2	44	29	8	6	1	2	0	2	44	46
121	5	34	24	8	3	1	2	1	3	36	39
125	6	32	20	11	3	1	1	2	3	35	38
123	5	28	23	5	2	0	3	0	3	30	33
135	4	40	24	14	4	1	1	0	1	43	44
136	5	37	33	5	2	0	1	1	2	40	42
	311	1643	703	284	124	707	80	56	136	1818	1954

Online resource table 6. Top 50 DEGs between SWNTS-SWNs versus NS-SWNs

rownames(result1)	logFC	AveExpr	t	P.Value	adj.P.Val
MRPL12	-1.295840447	5.399584615	-10.14546118	6.09E-15	1.05E-10
H3-3A	0.894150407	9.832830769	10.00582628	1.05E-14	1.05E-10
MZT2B	-2.027160569	8.074753846	-9.741599188	2.99E-14	1.72E-10
MRPL41	-1.715065041	8.008476923	-9.687996306	3.69E-14	1.72E-10
MIF	-1.837655488	6.355261538	-9.645845326	4.37E-14	1.72E-10
NME3	-2.017502033	7.334661538	-9.603497431	5.17E-14	1.72E-10
PSMC1	0.70246748	10.75673846	9.285247482	1.83E-13	5.22E-10
TSPO	-1.744666667	9.010815385	-9.050472161	4.70E-13	1.10E-09
KLHL17	-1.257163618	6.600938462	-9.029765302	5.10E-13	1.10E-09
JOSD2	-1.937546748	7.314061538	-9.009701235	5.53E-13	1.10E-09
EVA1B	-1.64745122	6.660661538	-8.956775677	6.84E-13	1.12E-09
NOXA1	-1.752950203	6.768415385	-8.943209958	7.22E-13	1.12E-09
C1orf159	-1.438025407	7.009353846	-8.941382201	7.28E-13	1.12E-09
ZNF649	1.045964431	9.426446154	8.884409155	9.15E-13	1.30E-09
SCNN1D	-1.531081301	7.246092308	-8.84567566	1.07E-12	1.42E-09
ARSA	-1.45061687	8.869046154	-8.823813586	1.17E-12	1.45E-09
CAPN15	-1.373416667	8.133892308	-8.762559854	1.49E-12	1.68E-09

SENP3	-0.68102439	8.008569231	-8.739172613	1.64E-12	1.68E-09
NDUFS7	-1.687434959	8.350215385	-8.737817162	1.65E-12	1.68E-09
TPGS1	-1.182539634	6.577784615	-8.732684649	1.69E-12	1.68E-09
CCDC85B	-1.496886179	6.487523077	-8.677453168	2.11E-12	1.96E-09
TMUB1	-1.253568089	7.128753846	-8.670950523	2.16E-12	1.96E-09
LZTR1	-1.330223577	8.727230769	-8.654690255	2.31E-12	1.97E-09
HEXD	-1.193273374	8.474138462	-8.641194264	2.44E-12	1.97E-09
TRAPPC6A	-1.114963415	7.235784615	-8.621107995	2.64E-12	1.97E-09
MXD3	-1.174586382	6.614184615	-8.619099499	2.66E-12	1.97E-09
MFSD3	-1.13435061	6.663138462	-8.618043784	2.68E-12	1.97E-09
FBXL15	-1.304639228	6.845384615	-8.603331066	2.84E-12	1.99E-09
KIFC2	-1.590034553	8.398861538	-8.598171291	2.90E-12	1.99E-09
TCIRG1	-1.831555894	9.154830769	-8.563884458	3.33E-12	2.18E-09
PCSK4	-1.096653455	5.904276923	-8.559154887	3.39E-12	2.18E-09
PABPN1	-0.983788618	10.48287692	-8.537633974	3.70E-12	2.31E-09
SCAND1	-1.764531504	7.005553846	-8.518479395	4.00E-12	2.42E-09
MIB2	-1.664980691	8.069676923	-8.510731592	4.13E-12	2.42E-09
ALKBH7	-1.428457317	7.329276923	-8.492696986	4.44E-12	2.53E-09
ABHD8	-1.189502033	7.066384615	-8.455661007	5.16E-12	2.81E-09
ZNF787	-0.986143293	7.395153846	-8.452384339	5.23E-12	2.81E-09
REX1BD	-1.435121951	7.860230769	-8.432033082	5.67E-12	2.97E-09
SIRT6	-1.084534553	7.450507692	-8.400604237	6.44E-12	3.29E-09
LMF2	-1.718172764	8.820353846	-8.38358086	6.90E-12	3.44E-09
ZNHIT2	-1.057114837	6.075753846	-8.371046775	7.26E-12	3.53E-09
RABAC1	-1.333533537	7.008276923	-8.361813406	7.54E-12	3.58E-09
AP5Z1	-1.453529472	8.3368	-8.347653753	7.98E-12	3.70E-09
ANTKMT	-1.432634146	6.757661538	-8.333492129	8.45E-12	3.78E-09
CEBPD	-1.521776423	10.27272308	-8.33092324	8.54E-12	3.78E-09
SSBP4	-1.270667683	8.385123077	-8.325784915	8.72E-12	3.78E-09
ARRDC1	-1.294432927	7.733738462	-8.30838258	9.36E-12	3.97E-09
BRAT1	-1.235107724	8.477984615	-8.299122335	9.71E-12	4.03E-09
RPS3A	0.931560976	13.6974	8.285268691	1.03E-11	4.18E-09

Online resource table 7. Fusions identified using RNAseq dataset

	Schwannomatosis		Schwannoma				
#FusionName	#Samples (n=24)	% SWNTS samples	#Samples (n=41)	% SWN samples	Predicted_effect	Difference in %	Fusion_finding_method
NAIP--OCLN	16	66.67	12	29.27	out-of-frame	37.4	BOWTIE
NAIP--OCLN	1	4.17	1	2.44	in-frame	1.73	BOWTIE
PPIP5K1--CATSPER2	5	20.83	6	14.63	in-frame	6.2	BOWTIE
SAMD5--SASH1	7	29.17	2	4.88	in-frame	24.29	BOWTIE

CTSC--RAB38	12	50	14	34.15	in-frame	15.85	BOWTIE
AZGP1--GJC3	12	50	20	48.78	in-frame	1.22	BOWTIE;BOWTIE+STAR
SLC29A1--HSP90AB1	2	8.33	1	2.44	in-frame	5.89	BOWTIE
HDAC8--CITED1	1	4.17	2	4.88	in-frame	-0.71	BOWTIE
KANSL1--ARL17A	10	41.67	13	31.71	out-of-frame	9.96	BOWTIE
KANSL1--ARL17B	10	41.67	13	31.71	out-of-frame	9.96	BOWTIE

12
